# Transcriptional Approach for Decoding the Mechanism of *rpoC* Compensatory Mutations for the Fitness Cost in Rifampicin-Resistant *Mycobacterium tuberculosis*

**DOI:** 10.3389/fmicb.2018.02895

**Published:** 2018-11-30

**Authors:** Zhihong Xu, Aiping Zhou, Jiawei Wu, Aiwu Zhou, Jun Li, Shulin Zhang, Wenjuan Wu, Petros C. Karakousis, Yu-Feng Yao

**Affiliations:** ^1^Laboratory of Bacterial Pathogenesis, Department of Microbiology and Immunology, Shanghai Jiao Tong University School of Medicine, Shanghai, China; ^2^Department of Laboratory Medicine, Shanghai East Hospital, Tongji University School of Medicine, Shanghai, China; ^3^Faculty of Basic Medicine, Key Laboratory of Cell Differentiation and Apoptosis of the Chinese Ministry of Education, Shanghai Jiao Tong University School of Medicine, Shanghai, China; ^4^College of Biotechnology and Bioengineering, Zhejiang University of Technology, Hangzhou, China; ^5^Department of Medicine, Center for Tuberculosis Research, Johns Hopkins University School of Medicine, Baltimore, MD, United States

**Keywords:** *Mycobacterium tuberculosis*, rifampicin resistance, fitness cost, *rpoC* mutation, RNA sequence

## Abstract

Multidrug-resistant tuberculosis (TB), defined as TB resistant to the two first-line drugs, isoniazid and rifampin, is a serious challenge to global TB eradication efforts. Although mutations in *rpoA* or *rpoC* have been proposed to compensate for this fitness cost due to *rpoB* mutation in rifampicin-resistant *Mycobacterium tuberculosis* mutants, whether the compensatory effect exists and the underlying mechanisms of compensation remain unclear. Here, we used RNA sequencing to investigate the global transcriptional profiles of 6 rifampin-resistant clinical isolates with either single mutation in *rpoB* or dual mutations in *rpoB*/*rpoC*, as well as 3 rifampin-susceptible clinical isolates, trying to prove the potential compensatory effect of *rpoC* by transcriptomic alteration. In rifampin-free conditions, *rpoC* mutation was associated with *M. tuberculosis* upregulation of ribosomal protein-coding genes, dysregulation of growth-related essential genes and balancing the expression of arginine and glutamate synthesis-associated genes. Upon rifampin exposure of *M. tuberculosis* isolates, *rpoC* mutations were associated with the upregulation of the oxidative phosphorylation machinery, which was inhibited in the *rpoB* single mutants, as well as stabilization of the expression of rifampin-regulated essential genes and balancing the expression of genes involved in metabolism of sulfur-containing amino acids. Taken together, our data suggest that *rpoC* mutation may compensate for the fitness defect of rifampicin-resistant *M. tuberculosis* by altering gene expression in response to rifampin exposure.

## Introduction

Tuberculosis (TB) is an infectious disease caused by *Mycobacterium tuberculosis* and remains a major global health problem due to its high burden and death worldwide. Multidrug-resistant TB (MDR TB), defined as TB resistant to isoniazid and rifampin, is a continuing threat, with an estimated 600,000 new cases that were resistant to rifampin, the most effective first-line anti-TB drug (Vasilyeva et al., 2017).

Rifampin kills *M. tuberculosis* by binding to the β subunit of RNA polymerase, encoded by *rpoB*, where it exerts an early block of elongation of 2–3 nucleotide-long short RNA transcripts, thus inhibiting transcription (Campbell et al., 2001). Resistance to rifampin is primarily conferred by mutations in *rpoB*, which alter drug binding. Over 95% of rifampin-resistant *M. tuberculosis* clinical isolates harbor a mutation within the 81-bp region of *rpoB* known as the rifampin resistance-determining region (RRDR) (Ramaswamy and Musser, 1998), among which the most commonly observed mutations are S450L and H445Y, leading to high-level rifampin resistance (Mokrousov, 2004; Sandgren et al., 2009).

Given the essentiality of RNA polymerase for bacterial transcriptional processes, it stands to reason that mutations in *rpoB* could have profound effects on *M. tuberculosis* physiology or pathogenesis. Previous studies have reported that even in the absence of drug pressure, *rpoB* mutations confer a “fitness cost” in laboratory-derived strains of *M. tuberculosis*, including reduced growth rate and virulence compared to their drug-susceptible counterparts (Lloyd, 1987; Mariam et al., 2004; Gagneux et al., 2006; Rifat et al., 2017). However, Gagneux et al. (2006) reported previously that the same resistance mutations in *rpoB* are not necessarily associated with a fitness cost in *M. tuberculosis* clinical isolates, perhaps due to the presence of compensatory mutations, as have been reported in rifampin-resistant *Escherichia coli* (Reynolds, 2000). Specifically, mutations in the *rpoC* gene, encoding the β’ subunit of RNA polymerase, were associated with altered transcript elongation rate and gene expression, leading to metabolic changes and increased growth rate in rifampin-resistant *E. coli* (Conrad et al., 2010). Up to one third of rifampin-resistant *M. tuberculosis rpoB* mutant strains were found to harbor mutations in *rpoC* or *rpoA*, encoding the RNA polymerase α subunit (Comas et al., 2011). Song et al. (2014) reported that *M. tuberculosis* RpoC F452L and V483G alleles restored the transcription efficiency of RNA polymerase bearing RpoB S450L mutation. Although these findings suggest potential compensatory effects of *rpoA* or *rpoC* mutations for fitness costs associated with *rpoB* mutations in *M. tuberculosis*, the compensatory effect needs to be validated and the underlying molecular mechanisms remain to be elucidated.

In this study, we used next-generation sequencing to characterize the genomes and transcriptomes (with and without rifampin exposure) of 6 rifampin-resistant clinical isolates of *M. tuberculosis*, including 3 isolates with a single mutation in the *rpoB* and 3 isolates harboring *rpoB* and *rpoC* dual mutations, as well as 3 rifampin-susceptible isolates as reference. A list of differentially expressed genes (DEGs) was generated by comparing *rpoB* single mutant or *rpoBC* mutant isolates *vs*. drug-susceptible isolates before or after rifampin exposure. Dysregulated genes were further filtered by removing those displaying identical transcriptional regulation between the *rpoB* and *rpoBC* groups, as well as genes with missense mutations in open reading frames. The resulting gene candidate list was categorized using Kyoto Encyclopedia of Genes and Genomes (KEGG) pathways (Ogata et al., 1999) to identify metabolic pathways potentially critical for restoration of *M. tuberculosis* fitness in the context of *rpoB* mutation.

## Materials and Methods

### Bacterial Culture Conditions

*Mycobacterium tuberculosis* isolates were collected from Suzhou Fifth People’s Hospital and Wuxi Fifth People’s Hospital between September 2013 and February 2014. Isolates were grown in Middlebrook 7H9 broth (Martin et al., 1989) supplemented with 10% Oleic acid-Albumin-Dextrose-Catalase, 0.5% glycerol, 0.05% Tween-80 at 37°C without shaking. Rifampin was added at 80 μg/mL.

### Antimicrobial Phenotypic Susceptibility Testing

Drug susceptibility testing of all the strains was performed as recommended by WHO/IUATLD (International union for tuberculosis and lung disease), as previously described (Ma et al., 2018), using the four first-line anti-TB drugs (ethambutol, isoniazid, rifampin, and streptomycin) and four second-line anti-TB drugs (amikacin, levofloxacin para-aminosalicylic acid, and prothionamide). The final concentrations of drugs in Lowenstein-Jensen media were as follows: isoniazid (ISN) 0.2 μg/mL, ethambutol (EMB) 2.0 μg/mL, rifampin (RIF) 40.0 μg/mL, streptomycin (STR) 4.0 μg/mL, amikacin (AMK) 100 μg/mL, levoflooxacin (LEV) 2.0 μg/mL, para-aminosalicylic acid (PA) 1.0 μg/mL, and prothionamide (PT) 25.0 μg/mL. The strain was considered to be resistant to the specific drug when the growth rate was >1% compared to the control group (without any drugs).

### Genotyping Procedures

Genomic DNA was extracted from *M. tuberculosis* isolates as previously described (Song et al., 2014). Identification of the Beijing family was performed for all the strains collected in this study using the RD105 deletion-targeted multiplex PCR (DTM-PCR) method (Chen et al., 2007). The strains with no RD105 region amplification were classified as Beijing genotype, while the others containing RD105 region were classified as non-Beijing genotype.

### Whole Genome Sequencing, SNP and INDEL Identification

*Mycobacterium tuberculosis* isolates were grown to mid-logarithmic phase, and DNA was isolated using Cetrimonium bromide as previously described (Song et al., 2014). Genome sequencing was performed by the Chinese National Human Genome Center in Shanghai using Illumina HiSeq 2500. Raw reads were aligned to the reference *M. tuberculosis* H37Rv genome (Camus et al., 2002) and assembled using Velvet version 1.2.03. Protein-coding genes were predicted using Glimmer version 3.02, while tRNA and rRNA were identified using tRNAscan-SE version 2.0 and RNAmmer version 1.2, respectively. Pairwise-alignment according to the reference *M. tuberculosis* H37Rv genome was performed using R package Biostrings version 2.46.0 to identify single nucleotide polymorphisms (SNPs) and insertion/deletions (INDELs). All genome sequence data could be accessible in NCBI BioProject database (Accession No. PRJNA497952, PRJNA497955, PRJNA497954, PRJNA497944, PRJNA497947, PRJNA497946, PRJNA497950, PRJNA497943, and PRJNA497939).

### Molecular Modeling

The atomic model of the *M. tuberculosis* RNA polymerase transcription initiation complex (TIC) was used for modeling (RCSB accession number: 6C04) (Boyaci et al., 2018). The mutations I491V, G594E, and A734V were then mapped onto the three-dimensional structure of the *M. tuberculosis* RNA polymerase and visualized using PyMOL version 2.1.

### RNA Isolation and RNA Sequencing

*Mycobacterium tuberculosis* isolates were grown to mid-logarithmic phase in the presence or absence of rifampin as indicated. Total RNA extraction was performed using the RiboPure-Bacteria Kit (Ambion) according to the manufacturer’s recommendations with modifications (Ren et al., 2016). RNA sequencing was performed on an Illumina HiSeq 2500 using the NEBNext Ultra RNA Library Prep Kit. Unsupervised hierarchical clustering and heatmap representation of genome-wide expression profiles of 9 clinical isolates by RNA-Seq were performed in R package gplots using Reads Per Kilobase per Million mapped reads (RPKM). Differential gene expression analysis was performed using R package DEGseq version 1.32.0 and processed using MA-plot-based method with random sampling model. RNA-Seq raw data were deposited in NCBI Sequence Read Archive (SRA) database (Accession No. SRR8149449, SRR8149447, SRR8149496, SRR8149483, SRR8149500, SRR8149502, SRR8149437, SRR8149444, SRR8149498, SRR8149478, and SRR8149497).

### Construction of Weighted Gene Co-expression Network

To ensure that the results of network construction were reliable, outlier samples were removed. An appropriate soft threshold power was selected in accordance with standard scale-free networks, with which adjacencies between all differential genes were calculated by a power function. Then, the adjacency was transformed into a topological overlap matrix (TOM), and the corresponding dissimilarity (1-TOM) was calculated. Module identification was accomplished with the dynamic tree cut method by hierarchically clustering genes using 1-TOM as the distance measure with a deepsplit value of 2 and a minimum size cutoff of 30 for the resulting dendrogram. Highly similar modules were identified by clustering and then merged together with a height cut-off of 0.42. To test the stability of each identified module, module preservation and quality statistics were computed with the module preservation function implemented in the R package WGCNA.

### Statistical Analysis

Statistical analysis of data was performed using GraphPad Prism version 6. Data were presented as mean values with SD. DEGs were identified with a Benjamini-Hochberg FDR < 0.05. Functional enrichment analysis of DEGs was performed according to KEGG and visualized using R package clusterProfiler version 3.6.0. Cutoff of *P*-value was set to 1 to obtain all the annotated information in the KEGG database. Results were visualized by barplot or dotplot. Statistical significant differences was determined by Student’s *t*-test or two-way ANOVA among different groups. *P*-values < 0.05 denoted statistical significance.

### Ethics Statement

The study was approved by the ethical review boards of Suzhou Fifth People’s Hospital and Wuxi Fifth People’s Hospital.

## Results

### Drug-Resistance Profiles and Genomic Sequencing Data of Rifampin-Resistant *M. tuberculosis* Clinical Isolates

Initially, 16 *M. tuberculosis* clinical isolates were evaluated for inclusion in this study. In order to gain primary data on these isolates and optimize group matching, antimicrobial phenotypic susceptibility testing, genotyping and rifampin resistance-associated gene sequencing were performed. As shown in Supplementary Table S1, susceptibility tests toward first-line and second-line anti-TB agents identified 9 rifampin-susceptible isolates and 7 rifampicin-resistant isolates. Genotyping (Supplementary Figure S1) revealed 11 Beijing family isolates and 5 non-Beijing family isolates. Sequence analyses of the *rpoA, rpoB* and *rpoC* genes for each isolate are listed in Supplementary Table S2. To optimize the paired design, isolates were classified into three groups, rifampin-resistant isolates with *rpoB* single mutation, rifampin-resistant isolates with *rpoB*/*rpoC* (*rpoBC*) double mutations and rifampin-susceptible isolates. To balance the sample size, three isolates were assigned to each group. The genotype of each isolate was also screened to ensure that both Beijing and non-Beijing genotype would be included in each group. Consequently, 6 rifampin-resistant isolates (3 *rpoB* single mutant and 3 *rpoBC* mutant isolates) and 3 rifampin-susceptible isolates were selected and subjected to genome sequencing. Characteristics, genome sequencing information and predicted ORFs of these 9 isolates are summarized in Table 1 and Supplementary Tables S3, S4, respectively.

**Table 1:** Characteristics of clinical *M. tuberculosis* isolates used for this study.

Isolates	Mutations		Family	Drug-resistance profiles							
	RpoB	RpoC		ISN	EMB	RIF	STR	AMK	LEV	PA	PT
sz9610	H445Y, P454H	None	B	S	S	R	S	S	S	S	S
wx2	H445Y	None	B	S	S	R	S	S	S	S	S
wh18	S450L	None	NB	S	S	R	S	S	S	S	S
sz6213	S450L	A734V	B	S	S	R	S	S	S	S	S
sz596	S450L	I491V	B	S	S	R	S	S	S	S	S
wx6	H445Y	G594E	NB	S	S	R	S	S	S	S	S
sz1	None	None	NB	S	S	S	S	S	S	S	S
sz3	None	None	B	S	S	S	S	S	S	S	S
sz6	None	None	NB	S	S	S	S	S	S	S	S

*S, susceptible; R, resistant; ISN, isoniazid; EMB, ethambutol; RIF, rifampicin; STR, streptomycin; AMK, amikacin; LEV, Levoflooxacin; PA, para-aminosalicylic acid; PT, prothionamide; B, Beijing; NB, non-Beijing*.

### Structural Analysis of *rpoC* Mutations in Rifampin-Resistant Isolates

**FIGURE 1:**
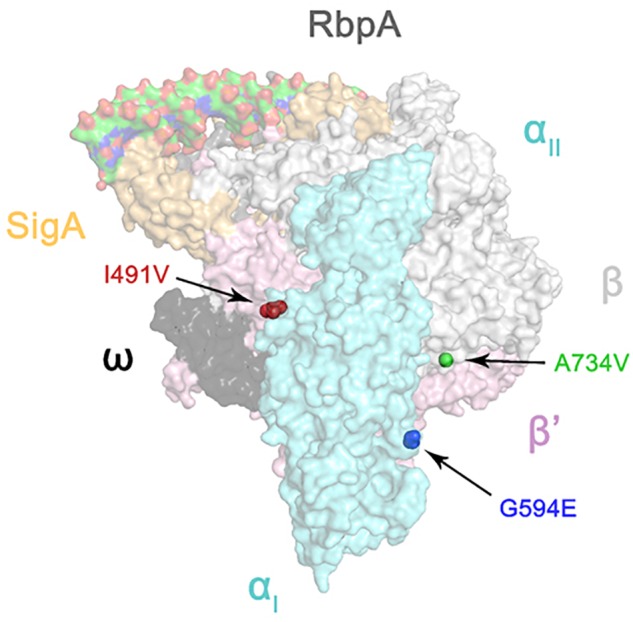
Three-dimensional representation of *rpoC* mutations on the atomic model of the *M. tuberculosis* RNA polymerase (represented by transparent molecular surfaces). The enzyme is depicted as α subunit (cyan), β subunit (white), β′ subunit (pink), ω subunit (black), SigA (yellow), and RbpA (gray). Compensatory mutations on β′ subunits are highlighted as red, blue, and green sphere pointed by arrow, respectively.

Since the RNA polymerase subunits RpoA, RpoB, and RpoC interact with each other (Luo et al., 2005), we hypothesized that non-synonymous polymorphisms in *rpoC* occurring in *rpoB* mutant isolates were likely to be compensatory (Zhang et al., 1999). We then mapped the three amino acid substitutions of RpoC onto the three-dimensional structure of the *M. tuberculosis* RNA polymerase (Boyaci et al., 2018). As shown in Figure 1, I491V, also reported by Comas et al. (2011), is located at the interface between the α and β’ subunits; A734V is near the interface between the β and β’ subunits; G594E maps to a region between the two α helices, and the substitution of glycine with glutamate changes the charge of the side chain from neutral to negative. Interestingly, Stefan et al. recently reported similar findings that F452L and V483G, two mutants of RpoC near I491V, displayed a compensatory effect on RpoB S450L mutated RNA polymerase *in vitro* by recovering the defects of open-promoter complex stability and elongation rate, enhancing termination efficiency and RNA primer hydrolysis (Stefan et al., 2018). Their work demonstrates the possibility of and underlying mechanism of mutations in RpoC compensating for functional defects of RNA polymerase resulting from mutations in RpoB. Therefore, we speculate that the 3 mutations we identified also probably affect the interaction between the subunits of RNA polymerase, which requires confirmation in future studies.

### Transcriptional Profiles of *M. tuberculosis* Rifampin-Resistant Isolates in Rifampin-Free Conditions

Since *rpoC* mutations alter the kinetic parameters of RNA polymerase in *E. coli* (Conrad et al., 2010), we hypothesized that *rpoC* mutations may similarly influence gene transcription in rifampin-resistant *M. tuberculosis*. To test this hypothesis, we performed RNA sequencing to determine the transcriptional profiles of the 9 clinical isolates in rifampin-free conditions.

We first performed unsupervised hierarchical clustering analysis to determine whether *rpoC* mutations affect the genome-wide expression profiles of rifampin-resistant isolates harboring *rpoBC* double mutations. The expression profiles of rifampin-resistant isolates harboring *rpoB* single mutation (sz9610, wx2, and wh18), rifampin-resistant isolates with *rpoBC* double mutations (sz6213, sz596, and wx6) and rifampin-susceptible isolates (sz1, sz3, and sz6) were subjected to clustering analysis and presented as heatmap using RPKM (Supplementary Figure S2). The expression profiles of 9 isolates were generally divided into two groups, *rpoBC* double mutants (sz596 and sz6213) and rifampin-susceptible isolates (sz1 and sz6) were clustered in one group, the other 5 isolates including *rpoB* single mutants (sz9610, wx2, and wh18), *rpoBC* double mutants (wx2) and rifampin-susceptible isolates (sz3) were clustered in the other group. These results suggest that *rpoBC* double mutants have more similar expression profiles to rifampin-susceptible isolates compared with *rpoB* single mutants.

Then differential gene expression analysis was performed for the following pairs: *rpoB* single mutant isolates vs. drug-susceptible isolates, and *rpoBC* isolates vs. drug-susceptible isolates. Supplementary Tables S5, S6 contains a summary of the dysregulated genes for each comparison.

To identify dysregulated genes associated with *rpoC* mutation, the genes were filtered using the following criteria: (1) Differential regulation in the comparison of *rpoB* single mutant isolates vs. drug-susceptible isolates and *rpoBC* isolates vs. drug-susceptible isolates; (2) Differential regulation with fold change ≥1.5 of at least one of 3 isolates in either the *rpoB* or *rpoBC* groups. A total of 474 genes that meet both criteria were subjected to further analyses.

*M. tuberculosis* has evolved significant genomic diversity, including multiple SNPs or INDELs, to adapt to the diverse environment encountered during natural infection of the human host (O’Neill et al., 2015). Therefore, to exclude the potential influence of genetic variation on transcription, comparative genomic analyses were performed to identify SNPs or INDELs in all 474 genes. These SNPs are listed in Supplementary Table S7. Genes with missense mutations were discarded. Finally, 360 DEGs between the *rpoB* and *rpoBC* groups were subjected to further study.

### Functional Categorization of *rpoC* Mutation-Associated Genes in Rifampin-Free Conditions

**FIGURE 2:**
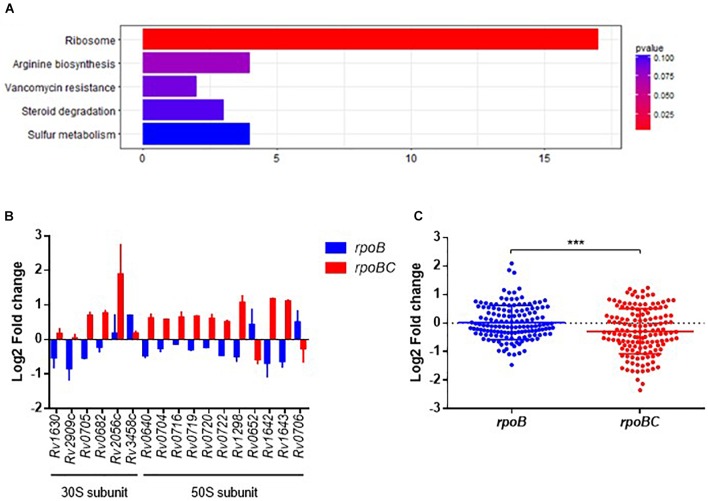
Global expression changes due to *rpoC* mutations in rifampicin-free conditions. **(A)** Top 5 KEGG enrichment of dysregulated genes. Genes that differentially regulated between comparisons of *rpoB*-mutated isolates vs. drug-susceptible isolates, *rpoBC*-mutated isolates vs. drug-susceptible isolates with fold change ≥1.5 of at least one of three isolates in either group remained. Genes with missense mutations were discarded. **(B)** Expression of dysregulated genes enriched in ribosome pathway. Fold change represents the average from two isolates in each group. **(C)** Fold change distribution of dysregulated essential genes due to *rpoC* mutations in rifampicin-free conditions. Dots represent the fold change of each gene from two isolates in each group. Student’s *t*-test, ^∗∗∗^*P* = 0.0002.

To explore the biological function of dysregulated genes associated with *rpoC* mutation, we performed KEGG enrichment analysis of the above 360 genes. 93 of these were enriched in 76 pathways (Supplementary Figure S3 and Figure 2A). The top 5 pathways represented include genes related to ribosome synthesis, arginine biosynthesis, vancomycin resistance, steroid degradation and sulfur metabolism.

The most significantly enriched pathway was related to ribosomes (FDR = 0.0005, Figure 2A). As shown in Figure 2B, 17 genes (six 30S subunit-coding genes, eleven 50S subunit-coding genes) are assigned to this pathway. For example, relative to rifampin-susceptible isolates, *Rv1630/rpsA* and *Rv2909c/rpsP* were downregulated in the *rpoB* group and *Rv0640/rplK, Rv0720/rplR, Rv0704/rplB, Rv0722/rpmD, Rv1298/rpmE, Rv0716/rplE*, and *Rv0719/rplF* were upregulated in the *rpoBC* group. *Rv1643/rplT* and *Rv1642/rpmI* showed reduced expression in the *rpoB* group but increased expression in the *rpoBC* group compared to rifampin-susceptible isolates.

Sassetti et al. have identified 830 genes that are required for the *in vitro* growth and *in vivo* survival of *M. tuberculosis* (Sassetti and Rubin, 2003; Sassetti et al., 2003). To explore the potential role of *rpoC* mutation in mycobacterial growth, we investigated whether expression of these growth-required essential genes is affected by *rpoC* mutation. A total of 72 essential genes were differentially regulated in either the *rpoB* or *rpoBC* groups compared to rifampin-susceptible isolates (Supplementary Figure S4). Specifically, 20 genes were significantly upregulated and 9 genes were significantly downregulated in the *rpoB* group relative to rifampin-susceptible isolates, and 15 genes were significantly upregulated and 31 genes were significantly downregulated in the *rpoBC* group (Figure 2C).

### Co-expression Network of *rpoC* Mutation-Associated Genes in Rifampin-Free Conditions

**FIGURE 3:**
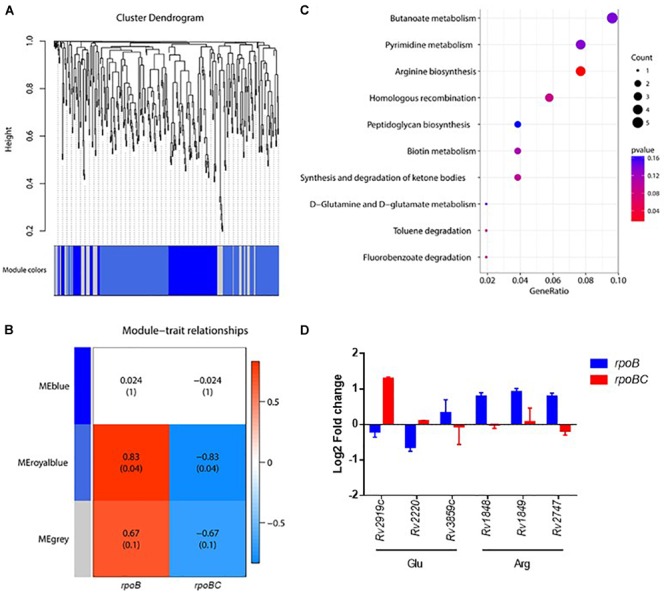
Co-expression networks of dysregulated genes due to *rpoC* mutations in rifampicin-free conditions. **(A)** Dendrogram of all differentially expressed genes clustered. Gene clustering tree (dendrogram) obtained by hierarchical clustering of adjacency-based dissimilarity. The colored row below the dendrogram indicates module membership identified by the dynamic tree cut method. Three kinds of color present three modules. **(B)** Correlation between each clustered module and mutation. Correlation index and *P*-value (parenthesized) are listed. Heatmap of the correlation between module eigengenes (MEs) and different group information (*rpoB* or *rpoBC*). **(C)** KEGG pathway enrichment analyses for genes in the royalblue module. The *x*-axis shows the ratio number of genes and the *y*-axis shows the KEGG pathway terms. The *P*-value of each term is colored according to the legend. **(D)** Expression of genes in glutamate and arginine synthesis. Fold change represents the average from two isolates in each group.

To further screen for key dysregulated genes associated with *rpoC* mutation, a weighted gene co-expression network analysis (WGCNA) was applied to construct the gene co-expression networks among 360 genes differentially regulated in the 6 rifampin-resistant isolates. As shown in Figure 3A, we identified three co-expressed modules (110 genes in blue, 195 genes in royalblue and 55 genes in gray modules) within which genes displayed a similar expression pattern. Among these modules, dysregulation of genes within the royalblue module showed the closest relation to *rpoB* (*R* = 0.83, *P* = 0.04) and *rpoBC* (*R* = -0.83, *P* = 0.04) mutation isolates (Figure 3B). KEGG enrichment analysis was then performed on 195 genes in the royalblue module. The top 5 pathways represented include arginine biosynthesis, homologous recombination, fluorobenzoate degradation, toluene degradation, and ketone body synthesis and degradation (Figure 3C).

The arginine biosynthesis pathway was significantly enriched (*P* = 0.0114) (Figure 3D). *Rv1848/ureA* (encoding the urease γ subunit), *Rv1849/ureB* (encoding the urease β subunit), *Rv2747/argA* (encoding an N-acetylglutamate synthase) were upregulated in the *rpoB* group. *Rv2220/glnA1* (encoding a glutamine synthetase) was downregulated in the *rpoB* group. These results suggest that *rpoC* mutation is responsible for stabilizing the expression of arginine synthesis-associated genes. Since arginine synthesis begins with the N-acetylation of L-glutamate in prokaryotes (Glansdorff and Xu, 2006), we therefore investigated whether genes involved in the synthesis of its precursor L-glutamate were also differentially regulated. As expected, *Rv2919c/glnK* (encoding a nitrogen assimilation regulatory protein, inhibiting ammonium uptake) was downregulated in the *rpoB* group but upregulated in the *rpoBC* group. *Rv3859c/gltB* (encoding the subunit of glutamine oxoglutarate aminotransferase for glutamate synthesis) was upregulated in the *rpoB* group (Figure 3D).

### Rifampin-Induced Transcriptional Profiles in Rifampin-Resistant Isolates

Since *M. tuberculosis* gene expression is altered following exposure to rifampin (Campbell et al., 2001), we next identified genes whose expression were affected by rifampin in the *rpoB* group but were reversed to homeostasis due to the presence of *rpoC* mutation. Differential gene expression analysis was performed pair-wise, i.e., pre-exposure vs. post-exposure of *rpoB* single mutant isolates and *rpoBC* mutant isolates, respectively. The numbers of dysregulated genes are summarized in Supplementary Tables S8, S9. To identify genes whose expression is dysregulated by *rpoC* mutation, the genes were further filtered using the same criteria as used for analysis in rifampin-free conditions. A total of 435 differentially regulated genes were identified. SNPs of these genes are listed in Supplementary Table S10.

### Functional Categorization of *rpoC* Mutation-Associated Genes Responsive to Rifampin

**FIGURE 4:**
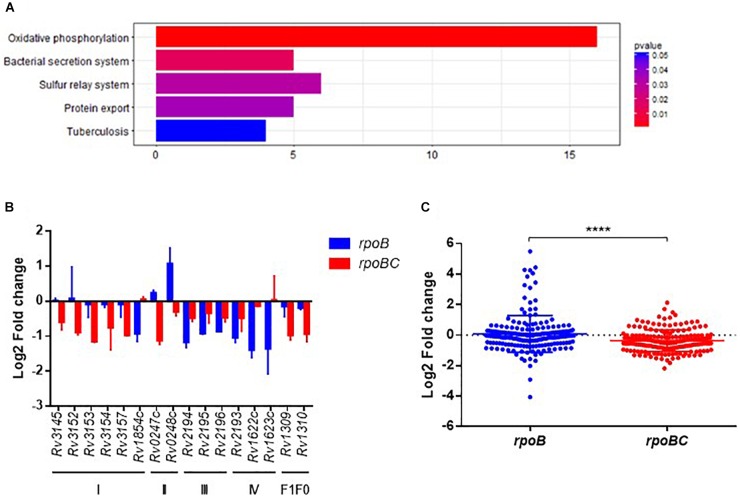
Global expression changes due to *rpoC* mutations after rifampicin stimulation. **(A)** Top 5 KEGG enrichment of dysregulated genes. Genes that differentially regulated between comparisons of pre-stimulation vs. post-stimulation of *rpoB*-mutated isolates and *rpoBC*-mutated isolates with fold change ≥1.5 of at least one of three isolates in either group remained. Genes with missense mutations were discarded. **(B)** Expression of dysregulated genes enriched in oxidative phosphorylation pathway. Fold change represents the average from two isolates in each group. **(C)** Fold change distribution of dysregulated essential genes due to *rpoC* mutations after rifampicin stimulation. Dots represent the fold change of each gene from two isolates in each group. ^∗∗∗∗^*p* < 0.01.

KEGG enrichment analysis was then applied to obtain the functional annotation of these dysregulated genes. As a result, 120 of 435 candidates were enriched in 76 pathways (Figure 4A and Supplementary Figure S5). The top 5 pathways represented include oxidative phosphorylation, bacterial secretion systems, sulfur relay systems, protein export, and pathways involved in TB pathogenesis and host immune responses.

The Oxidative phosphorylation pathway was significantly enriched (FDR = 0.0016, Figure 4A). As demonstrated in Figure 4B, 16 genes are categorized to this pathway and can be sorted into several subsets of oxidative phosphorylation machinery. For example, in complex I, *Rv3145/nuoA, Rv3152/nuoH, Rv3153/nuoI, Rv3154/nuoJ, Rv3157/nuoM* [encoding a type I NADH dehydrogenase (Weinstein et al., 2005)] were downregulated in *rpoBC* group. *Rv1854c/ndh* [encoding a type II NADH dehydrogenase (Weinstein et al., 2005)] was downregulated in *rpoB* group.

Among the essential genes (Sassetti and Rubin, 2003; Sassetti et al., 2003), 91 genes were differentially expressed between the *rpoB* and *rpoBC* groups (Supplementary Figure S6). The global transcription profiles of the two groups are significantly different (Figure 4C). Thus, 14 genes were upregulated and 21 genes were downregulated in the *rpoB* group. 12 genes were upregulated and 47 genes were downregulated in the *rpoBC* group. However, the fold changes in expression of dysregulated genes in the *rpoBC* group are smaller than those in the *rpoB* group.

### Co-expression Networks of *rpoC* Mutation-Associated Genes Responsive to Rifampin

**FIGURE 5:**
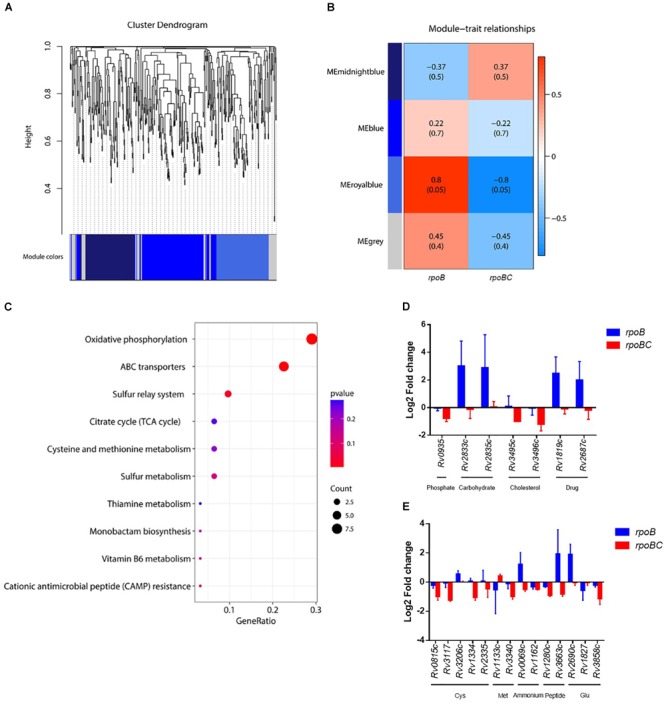
Co-expression networks of dysregulated genes due to *rpoC* mutations after rifampicin stimulation. **(A)** Dendrogram of all differentially expressed genes clustered. Gene clustering tree (dendrogram) obtained by hierarchical clustering of adjacency-based dissimilarity. The colored row below the dendrogram indicates module membership identified by the dynamic tree cut method. Four kinds of color present four modules. **(B)** Correlation between each clustered module and mutation. Correlation index and *P*-value (parenthesized) are listed. Heatmap of the correlation between module eigengenes (MEs) and different group information (*rpoB* or *rpoBC*). **(C)** KEGG pathway enrichment analyses for genes in the royalblue module. The *x*-axis shows the ratio number of genes and the *y*-axis shows the KEGG pathway terms. The *P*-value of each term is colored according to the legend. **(D)** Expression of genes coding ABC transporters. Fold change represents the average from two isolates in each group. **(E)** Expression of genes in cysteine and methionine metabolism. Fold change represents the average from two isolates in each group.

Key dysregulated genes associated with *rpoC* mutation were identified through gene construction of co-expression networks based on expression of 435 candidates. As shown in Figures 4, 5A co-expressed modules (107 genes in midnightblue, 163 genes in blue, 113 genes in royalblue and 52 genes in gray module) were identified. Dysregulated genes within the royalblue module showed the closest relation in *rpoB* (*R* = 0.8, *P* = 0.05) and *rpoBC* (*R* = -0.8, *P* = 0.05) mutant isolates (Figure 5B). The top 5 pathways emerging from KEGG enrichment analysis of the 113 genes in the royalblue module were oxidative phosphorylation, ABC transporters, sulfur relay system, cationic antimicrobial peptide resistance, and sulfur metabolism (Figure 5C).

Several ABC transporters (Figure 5D) were found to be dysregulated in each mutant group. For example, *Rv0935/pstC1* (encoding the subunit of an ABC transporter for phosphate uptake) was downregulated in the *rpoBC* group. *Rv2833c/ugpB* and *Rv2835c/ugpA* (encoding the subunits of an ABC transporter for carbohydrate uptake) were upregulated in the *rpoB* group. *Rv3495c/lprN* and *Rv3496c/mce4D* (encoding the subunits of an ABC transporter for cholesterol uptake) were downregulated in the *rpoBC* group. *Rv1819c* and *Rv2687c* (each encoding the subunit of a drug efflux pump) were upregulated in the *rpoB* group.

In the sulfur relay system pathway (Figure 5E), *Rv0815c/cysA2* and *Rv3117/cysA3* (each encoding a thiosulfate sulfurtransferase) were downregulated in the *rpoBC* group. *Rv3206c/moeZ* (encoding an adenylyltransferase) was upregulated in the *rpoB* group. Considering that proteins encoded by these genes are involved in sulfur assimilation, their reduced transcription levels in the *rpoBC* group compared to the *rpoB* group suggest that *rpoC* mutation might reduce sulfur assimilation following rifampin exposure.

Sulfur is mainly assimilated to synthesize cysteine and methionine in mycobacteria (Pinto et al., 2007). We therefore investigated whether genes in their synthesis were also dysregulated in the *rpoB* and *rpoBC* mutant isolates. As shown in Figure 5E, in the cysteine synthesis pathway, *Rv1334/mec* (encoding a Zn^2+^-dependent hydrolase) and *Rv2335/cysE* (encoding a serine acetyltransferase) were downregulated in *rpoBC* isolates, suggesting reduced cysteine synthesis in this group. In the methionine synthesis pathway, *Rv1133c/metE* (encoding a methionine synthase) was upregulated in the *rpoBC* isolates. On the other hand, *Rv3340/metC* (encoding a cystathionine β-lyase) was downregulated in the *rpoBC* isolates. Taken together, these results indicate an increase in methionine synthesis and consumption of homocysteine in *rpoBC* mutant isolates.

## Discussion

### *rpoC* Mutations Rectify the Interrupted Oxidative Phosphorylation Pathway

*Mycobacterium tuberculosis* is a highly aerobic bacterium that has adapted to inhabit a wide range of intracellular and extracellular environments (Cook et al., 2014). A fundamental feature of this adaptation is the utilization of variable sources for respiration and energy generation (Brodie and Gutnick, 1967). The inflow of electrons, the maintenance of proton motive force (PMF) and the synthesis of ATP, are essential for *M. tuberculosis* growth and survival (Sassetti et al., 2003; Tran and Cook, 2005; Rao et al., 2008). The transcription of the NDH-2 coding gene *Rv1854c/ndh* was significantly decreased in *rpoB* mutant isolates but remained stable in *rpoBC* mutant isolates following rifampin exposure. Considering the importance of NDH-2 activity for maintenance of the PMF, we propose that *rpoC* mutations might contribute to maintenance of PMF homeostasis by recovering the impaired expression of NDH-2. The inhibitor of succinate dehydrogenase, 3-nitropropionate, was able to dissipate membrane potential in *M. smegmatis*, and the transcription of the *sdh1* operon was upregulated in response to such high PMF conditions (Pecsi et al., 2014). We also observed higher mRNA levels of the *sdh1* operon, *Rv0248c/sdhA* and *Rv0247c/sdhB*, in the *rpoB* mutant isolates than in the *rpoBC* isolates, indicating that *rpoB* mutation might confer energy limitation in the absence of *rpoC* mutation.

Recent discoveries of small molecules targeting respiratory bc1 complex have triggered an interest in the cytochrome c pathway (Abrahams et al., 2012; Pethe et al., 2013; Arora et al., 2014; Rybniker et al., 2015). Our data show that the entire *qcrCAB* operon, together with another aa3-type cytochrome c oxidase coding gene, *Rv2193/ctaE*, were dramatically downregulated in *rpoB* single mutants, but less affected in *rpoBC* mutants following rifampin exposure. The impairment of the respiratory cytochrome c pathway suggests disrupted energy metabolism as a result of *rpoB* mutation, which might be rescued by compensatory *rpoC* mutations.

The gene encoding the alternative bd-type menaquinol oxidase displayed reduced expression in the *rpoB* single mutants. The role of this bd-type menaquinol oxidase (CydAB) is to protect *M. tuberculosis* from respiratory machinery inhibitors. Several studies in *M. tuberculosis* have demonstrated that inactivation of *cydA* made the bacilli hypersensitive to the ATP synthase inhibitor bedaquiline (Berney et al., 2014) and cytochrome c oxidoreductase inhibitors (Arora et al., 2014). Given the important role of CydAB for *M. tuberculosis* adaptation to stress conditions (Lu et al., 2015), the recovered expression of the *cydAB* operon resulting from *rpoC* mutations might compensate for the suppression in energy metabolism associated with *rpoB* mutation.

### *rpoC* Mutations Stabilize Amino Acid Metabolism

Amino acid metabolism in slow growing mycobacteria begins with the nitrogen assimilation pathways that incorporate ammonium or ammonia into glutamine and glutamate by glutamine synthetase and glutamine oxoglutarate aminotransferase (GOGAT) (Gillespie et al., 2011). Given that formation of glutamine from glutamate and ammonium is highly energy-consuming, as approximately 15% of the cell’s ATP supply is required (Reitzer, 2003), it is strictly regulated transcriptionally and/or post-translationally according to the cellular α-ketoglutarate/glutamine ratio (Gouzy et al., 2014). In the absence of rifampin exposure, *Rv2919c/glnK*, was slightly downregulated in the *rpoB* mutants, but dramatically upregulated in the *rpoBC* group. This suggests the constant demand for ammonium uptake observed in the *rpoB* group might decrease and provide a better intracellular nitrogen status in the *rpoBC* group. *Rv2220/glnA1* was downregulated while *Rv3859c/gltB* was upregulated in the *rpoB* group, which might consequently promote the conversion of glutamine to glutamate. Depletion of glutamine and imbalance of the glutamine/glutamate ratio might occur in *rpoB* single mutant isolates, thus affecting bacterial growth and viability, since prior evidence suggests that supplementation of culture medium with glutamine is more favorable than glutamate for the growth of *Mycobacterium avium* (McCarthy, 1978). A *M. tuberculosis* mutant deficient in *glnA1* became a glutamine auxotroph, requiring a relatively high level of exogenous L-glutamine for robust growth *in vitro* and exhibiting attenuated virulence *in vivo* (Tullius et al., 2003). Particularly, glutamine synthetase from pathogenic mycobacterial species, such as *M. tuberculosis* and *M. bovis*, was reported to be exported (Harth et al., 1994) and implicated in the synthesis of poly-L-glutamine-glutamate for maintaining the integrity of the mycobacterial cell wall (Harth et al., 2000). Inhibition of poly-L-glutamine-glutamate mRNA (Harth et al., 2000) or enzyme activity (Harth and Horwitz, 1999) reduced the growth of *M. tuberculosis* *in vitro*. Thus, we propose that downregulation of *glnA1* contributes to the growth defect observed in *rpoB* single mutant isolates, and this effect is reversed by compensatory *rpoC* mutations.

Although the expression of *Rv3858c/gltD* remained stable in *rpoB* single mutants following rifampin exposure, that of *Rv1827/garA*, the activator of GOGAT, was significantly downregulated. These data are consistent with decreased activity of GOGAT, perhaps leading to the reduction of glutamate synthesis. Since GarA also functions as an inhibitor of glutamate dehydrogenase and 2-ketoglutarate dehydrogenase, its decreased expression likely results in increased glutamate degradation and, in turn, the entry of α-ketoglutarate into the TCA cycle. Along with the suppression in energy metabolism we observed in *rpoB* single mutant isolates, this could compensate for a potential energy shortage. Moreover, the diminished synthesis and enhanced degradation of glutamate would continuously reduce intracellular glutamate concentrations. However, in the *rpoBC* mutant isolates, the situation was reversed. *Rv3858c/gltD* was downregulated while *Rv1827/garA* was upregulated, maintaining the homeostasis of glutamate synthesis and degradation. In addition to the ammonium assimilation pathway, another two genes are involved in ammonium production. *Rv0069c/sdaA* encodes a serine deaminase to release ammonium from serine (Gouzy et al., 2014). *Rv1162/narH* is located in the *narGHJI* operon encoding a nitrate reductase, which reduces nitrates to nitrites (Khan and Sarkar, 2012). The dysregulation of these genes in the two groups suggests their disparate requirements for ammonium. *Rv0069c/sdaA* was upregulated and *Rv1162/narH* remained unchanged in the *rpoB* single mutant isolates, while expression of *Rv0069c/sdaA* was unchanged and *Rv1162/narH* was downregulated in *rpoBC* mutant isolates. These results indicate a higher demand for available ammonium in the *rpoB* single mutant isolates than in the *rpoBC* mutant isolates. Finally, several genes encoding transporters associated with peptide or amino acid uptake, including *Rv1280c/oppA, Rv2690c*, and *Rv3663c/dppD* (Cole et al., 1998; Braibant et al., 2000; Green et al., 2000; Flores-Valdez et al., 2009), were found to be upregulated in the *rpoB* group but downregulated in the *rpoBC* group, suggesting potential defective *de novo* synthesis of amino acids caused by *rpoB* mutation, and a requirement for exogenous supplementation of amino acids.

Our work has several limitations. First, we were able to study only 3 isolates in each mutant group, which may explain the relatively high variability of transcriptional profiles within groups. Second, due to the high genetic heterogeneity and incomplete genomic sequencing of the 9 *M. tuberculosis* clinical isolates studied, genome-scale SNPs or INDELs may not be fully identified, complicating a thorough analysis of the potential influence of genetic variation on transcription.

In summary, we studied the compensatory effect of *rpoC* mutations on *rpoB* mutant *M. tuberculosis* using RNA sequencing, and found that *rpoC* mutations were associated with the transcriptional regulation of genes encoding ribosomal proteins, proteins essential for *M. tuberculosis* growth, components of the oxidative phosphorylation machinery and amino acid synthesis pathways. But our work still has several limitations. Firstly, the *M. tuberculosis* isolate number is inadequate and needs to be enlarged in further studies, as only 3 isolates were studied in each group, which may explain the high variation of transcriptional profiles even among isolates within the same group. Secondly, due to the high genetic heterogeneity and incomplete genomic sequencing of the 9 *M. tuberculosis* clinical isolates, genome-scale SNPs or INDELs will not be fully identified, which makes it difficult to thoroughly exclude the potential influence of genetic variation on transcription. Also, our findings need to be validated using isogenic *M. tuberculosis* strains in further studies due to the non-isogenicity of clinical strains. Therefore, future genetic and biochemical studies will focus on further characterizing the contribution of each of these pathways in restoring *rpoB* mutation-associated fitness costs in *M. tuberculosis* laboratory strains.

## Author Contributions

All authors listed have made a substantial, direct and intellectual contribution to the work, and approved it for publication.

## Conflict of Interest Statement

The authors declare that the research was conducted in the absence of any commercial or financial relationships that could be construed as a potential conflict of interest.
