# Supplementary material for: Transcriptional Approach for Decoding the Mechanism of rpoC Compensatory Mutations for the Fitness Cost in Rifampicin-Resistant Mycobacterium tuberculosis
Source: Front Microbiol. 2018 Nov 30;9:2895. doi: 10.3389/fmicb.2018.02895 (PMC6283890; doi:10.3389/fmicb.2018.02895)
Supplement: Supplementary file 1 [file Table_1.DOCX]

**Supplementary materials**

**Supplementary figures**

**Figure S1**


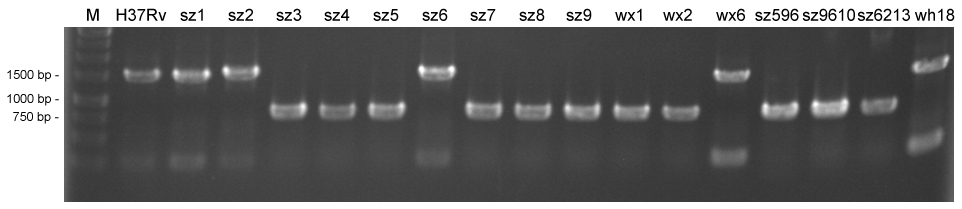


**Genotyping of 16 clinical isolates.** Amplification product of Beijing type is 761 bp in length and non-Beijing type is 1466 bp in length. M represents the DNA ladder.

**Figure S2**


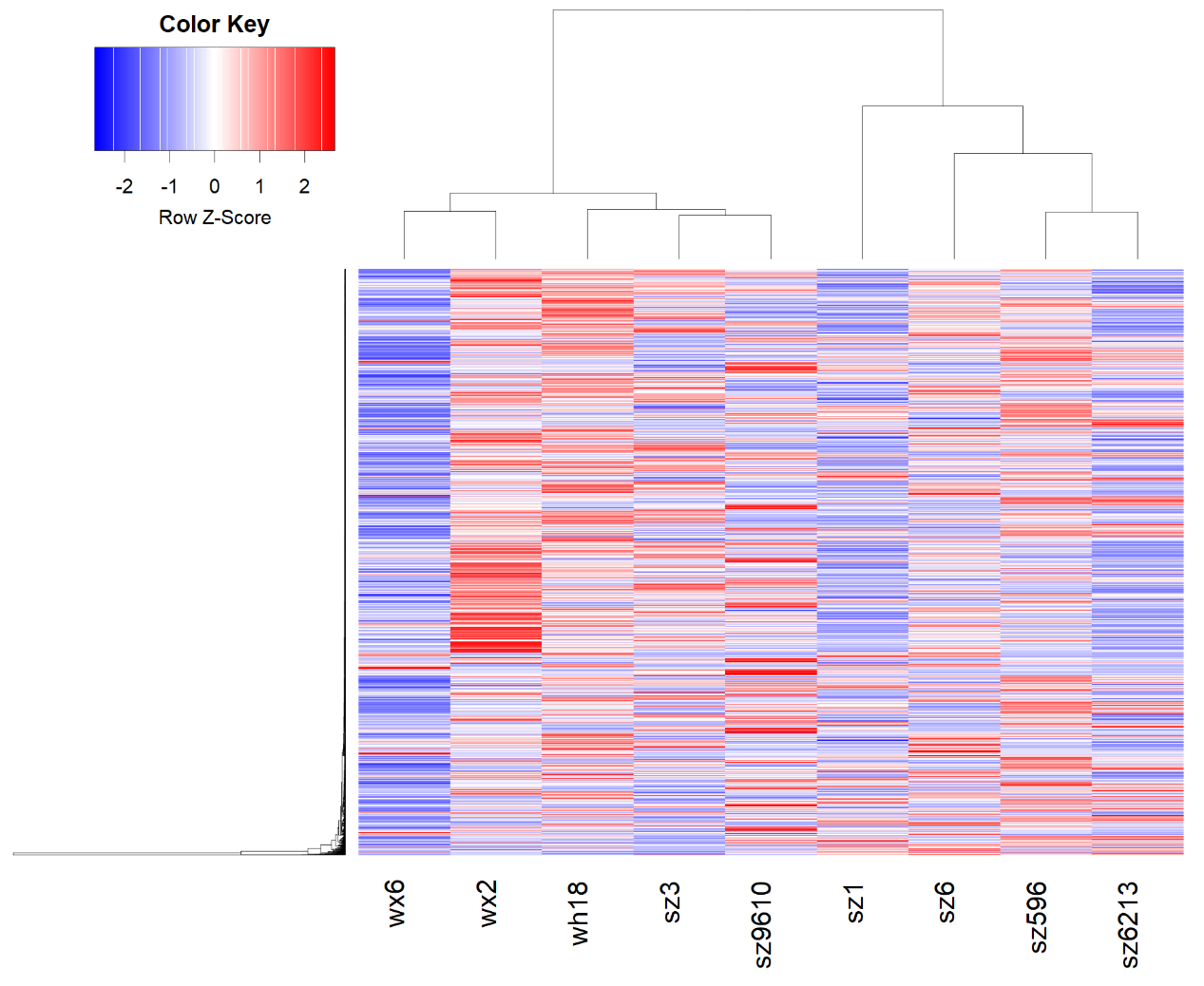


**Unsupervised hierarchical clustering and heatmap representation of genome-wide expression profiles of 9 clinical isolates** **by RNA-seq.** Expression profiles of rifampin-resistant isolates with *rpoB* single mutation (sz9610, wx2, wh18), rifampin-resistant isolates with *rpoB/rpoC* double mutations (sz6213, sz596, wx6) and rifampin-susceptible isolates (sz1, sz3, sz6) were subjected to clustering analysis and presented as heatmap using Reads Per Kilobase per Million mapped reads (RPKM). Down below shows the names of the isolates.

**Figure S3**

**
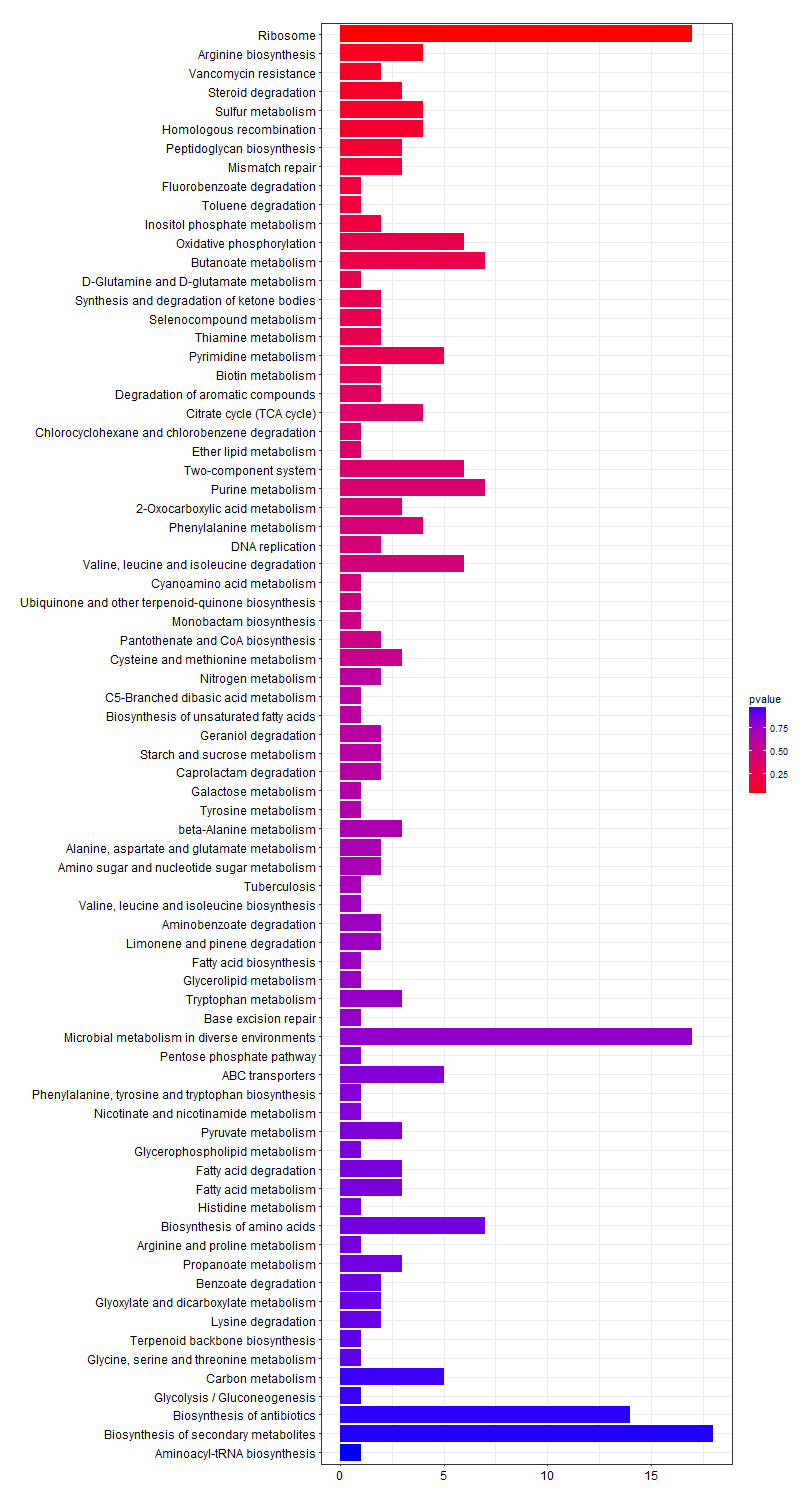
**

**KEGG enrichment of dysregulated gene expression due to *rpoC* mutations of *M. tuberculosis* in rifampin-free conditions.** Genes that differentially regulated between comparisons of *rpoB* mutated isolates vs drug-susceptible isolates, *rpoBC* mutated isolates vs drug-susceptible isolates with fold change ≥ 1.5 of at least one of 3 isolates in either group remained. Genes with missense mutations in ORFs were discarded.

**Figure S4**

**Dysregulated expression of *M. tuberculosis* essential genes in isolates containing *rpoB* or *rpoB/rpoC* mutations during growth in rifampin-free conditions.** Fold change represents the mean value + SD of gene expression from 2 isolates in each group relative to that of rifampin-susceptible isolates.

**Figure S5**


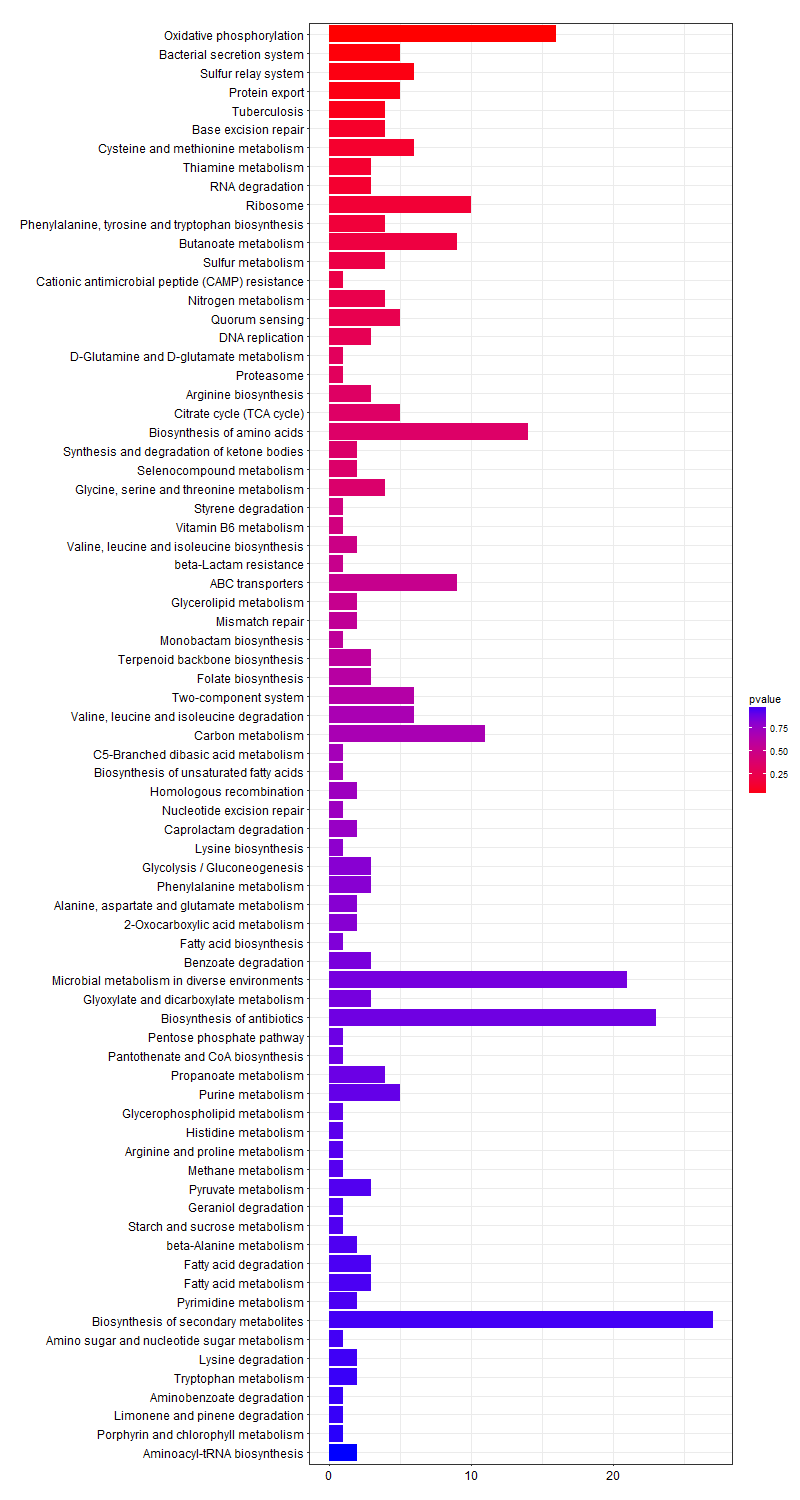


**KEGG enrichment of dysregulated genes due to *rpoC* mutations following rifampin exposure of *M. tuberculosis*.** Genes that differentially regulated between comparisons of pre-exposure vs. post-exposure of *rpoB* mutated isolates and *rpoBC* mutated isolates with fold change ≥ 1.5 of at least one of 3 isolates in either group remained. Genes with missense mutations in ORFs were discarded.

**Figure S6**

**Essential genes for *M. tuberculosis* growth are differentially expressed between the *rpoB* and *rpoBC* groups after exposure to rifampin.** Fold change represents the average from 2 isolates in each group. Fold change represents the mean value + SD of gene expression from 2 isolates in each group relative to that of rifampin-susceptible isolates.

**Supplementary tables**

Table S1 Drug-resistance profiles of 16 clinical *M. tuberculosis* isolates.

| **Isolates** | **Drug-resistance profile** | | | | | | | |
| --- | --- | --- | --- | --- | --- | --- | --- | --- |
|  | **ISN** | **EMB** | **RIF** | **STR** | **AMK** | **LEV** | **PA** | **PT** |
| sz1 | S | S | S | S | S | S | S | S |
| sz2 | S | S | S | S | S | S | S | S |
| sz3 | S | S | S | S | S | S | S | S |
| sz4 | S | S | S | S | S | S | S | S |
| sz5 | S | S | S | S | S | S | S | S |
| sz6 | S | S | S | S | S | S | S | S |
| sz7 | S | S | S | S | S | S | S | S |
| sz8 | S | S | S | S | S | S | S | S |
| sz9 | S | S | S | S | S | S | S | S |
| wx1 | S | S | R | S | S | S | S | S |
| wx2 | S | S | R | S | S | S | S | S |
| wx6 | S | S | R | S | S | S | S | S |
| sz596 | S | S | R | S | S | S | S | S |
| sz9610 | S | S | R | S | S | S | S | S |
| sz6213 | S | S | R | S | S | S | S | S |
| wh18 | S | S | R | S | S | S | S | S |

*M. tuberculosis* isolates were collected from Suzhou Fifth People's Hospital and Wuxi Fifth People's Hospital between September 2013 and February 2014. For antimicrobial phenotypic susceptibility testing, antibiotics were added at the following concentrations: isoniazid (ISN) 0.2 μg/mL, ethambutol (EMB) 2.0 μg/mL, rifampin (RIF) 40.0 μg/mL, streptomycin (STR) 4.0 μg/mL, amikacin (AMK) 100 μg/mL, levoﬂoxacin (LEV) 2.0 μg/mL, para-aminosalicylic acid (PA) 1.0 μg/mL, prothionamide (PT) 25.0 μg/mL. S, susceptible; R, resistant.

Table S2 Mutations in *rpoA*, *rpoB* or *rpoC* of 16 clinical *M. tuberculosis* isolates.

| **Isolates** | **Mutation** | | |
| --- | --- | --- | --- |
|  | ***rpoB*** | ***rpoA*** | ***rpoC*** |
| sz1 | none | none | none |
| sz2 | none | none | none |
| sz3 | none | none | none |
| sz4 | none | none | none |
| sz5 | none | none | none |
| sz6 | none | none | none |
| sz7 | none | none | none |
| sz8 | none | none | none |
| sz9 | none | none | none |
| wx1 | H445Y | none | none |
| wx2 | H445Y | none | none |
| wx6 | H445Y | none | G594E |
| sz596 | S450L | none | I491V |
| sz9610 | H445Y, P454H | none | none |
| sz6213 | S450L | none | A734V |
| wh18 | S450L | none | none |

H, Histidine; Y, Tyrosine; G, Glycine; E, Glutamate; S, Serine; L, Leucine; I, Isoleucine; V, Valine; P, Proline; A, Alanine.

Table S3 Genomic sequencing of 9 *M. tuberculosis* clinical isolates.

| **Isolates** | **Reads** | **Coverage (X)** | **Contigs** | | | | |
| --- | --- | --- | --- | --- | --- | --- | --- |
|  |  |  | **Count** | **Total length (bp)** | **Average length (bp)** | **Maximum length (bp)** | **N50 (bp)** |
| sz9610 | 4282162 | 299 | 179 | 4288720 | 24230 | 184044 | 55827 |
| wx2 | 4771972 | 332 | 158 | 4303547 | 27573 | 292394 | 58721 |
| wh18 | 3974360 | 276 | 167 | 4317408 | 25852 | 179920 | 57941 |
| sz6213 | 4740371 | 330 | 177 | 4308809 | 24477 | 183482 | 57326 |
| sz596 | 4915168 | 344 | 175 | 4285579 | 24489 | 198425 | 55891 |
| wx6 | 5031572 | 350 | 156 | 4312506 | 27644 | 245079 | 57941 |
| sz1 | 3599045 | 251 | 108 | 4349650 | 40274 | 263685 | 104078 |
| sz3 | 4496790 | 313 | 164 | 4300094 | 26376 | 211934 | 64003 |
| sz6 | 4193482 | 293 | 151 | 4287595 | 28579 | 184100 | 59852 |

Table S5 Number of dysregulated genes in each rifampin-resistant *M. tuberculosis* isolate cultured in rifampin-free conditions.

| **Isolates** | **Number of genes** | | | **Proportion of dysregulated genes** |
| --- | --- | --- | --- | --- |
|  | **Upregulated** | **Downregulated** | **Not differentially expressed** |  |
| wx2 | 1034 | 290 | 2445 | 35.13% |
| wh18 | 1004 | 230 | 2535 | 32.74% |
| sz9610 | 778 | 441 | 2550 | 32.34% |
| sz596 | 542 | 459 | 2768 | 26.56% |
| sz6213 | 413 | 1135 | 2221 | 41.07% |
| wx6 | 390 | 397 | 2982 | 20.88% |

Table S7 Summary of SNPs in dysregulated genes associated with *rpoC* mutations in *M. tuberculosis* rifampin-resistant isolates cultured in rifampicin-free conditions.

| **Synonym** | **Location** | **Reference** | | | **Open reading frame** | | |
| --- | --- | --- | --- | --- | --- | --- | --- |
|  |  | **Base** | **Codon** | **Amino acid** | **Base** | **Codon** | **Amino acid** |
| **wx2** | | | | | | | |
| *Rv0012* | 697 | T | TGC | Cys | C | CGC | Arg |
| *Rv0012* | 773 | G | GGC | Gly | T | GTC | Val |
| *Rv0013* | 204 | C | ATC | Ile | G | ATG | Met |
| *Rv0169* | 937 | T | TCC | Ser | G | GCC | Ala |
| *Rv0172* | 563 | T | ATC | Ile | C | ACC | Thr |
| *Rv0174* | 1109 | T | CTG | Leu | C | CCG | Pro |
| *Rv0192* | 379 | T | TCG | Ser | C | CCG | Pro |
| *Rv0221* | 63 | G | ATG | Met | A | ATA | Ile |
| *Rv0252* | 2323 | G | GTG | Val | T | TTG | Leu |
| *Rv0284* | 641 | C | CCG | Pro | G | CGG | Arg |
| *Rv0398c* | 87 | A | GAA | Glu | C | GAC | Asp |
| *Rv0530* | 692 | C | CCC | Pro | T | CTC | Leu |
| *Rv0576* | 698 | G | CGC | Arg | A | CAC | His |
| *Rv0577* | 220 | C | CCG | Pro | G | GCG | Ala |
| *Rv0667* | 1333 | C | CAC | His | T | TAC | Tyr |
| *Rv0697* | 1423 | G | GCC | Ala | C | CCC | Pro |
| *Rv0787* | 799 | T | TAC | Tyr | C | CAC | His |
| *Rv0792c* | 41 | G | CGG | Arg | A | CAG | Gln |
| *Rv0825c* | 203 | T | TTC | Phe | G | TGC | Cys |
| *Rv0844c* | 505 | G | GGT | Gly | C | CGT | Arg |
| *Rv1009* | 793 | G | GTG | Val | A | ATG | Met |
| *Rv1168c* | 500 | C | CCG | Pro | T | CTG | Leu |
| *Rv1207* | 217 | C | CGG | Arg | G | GGG | Gly |
| *Rv1224* | 22 | T | TGG | Trp | G | GGG | Gly |
| *Rv1317c* | 34 | A | ATC | Ile | G | GTC | Val |
| *Rv1477* | 221 | T | GTT | Val | C | GCT | Ala |
| *Rv1486c* | 594 | G | AAG | Lys | T | AAT | Asn |
| *Rv1592c* | 964 | A | ATT | Ile | G | GTT | Val |
| *Rv1795* | 703 | G | GTG | Val | A | ATG | Met |
| *Rv1815* | 247 | A | ATC | Ile | T | TTC | Phe |
| *Rv1821* | 1228 | G | GTG | Val | A | ATG | Met |
| *Rv1847* | 394 | C | CGG | Arg | T | TGG | Trp |
| *Rv1860* | 406 | T | TTC | Phe | C | CTC | Leu |
| *Rv1900c* | 612 | T | ATT | Ile | G | ATG | Met |
| *Rv2015c* | 453 | A | GAA | Glu | T | GAT | Asp |
| *Rv2057c* | 137 | G | CGT | Arg | T | CTT | Leu |
| *Rv2090* | 1074 | C | TTC | Phe | G | TTG | Leu |
| *Rv2351c* | 1336 | A | ACT | Thr | G | GCT | Ala |
| *Rv2409c* | 671 | C | ACC | Thr | A | AAC | Asn |
| *Rv2482c* | 2332 | T | TGC | Cys | C | CGC | Arg |
| *Rv2484c* | 398 | C | GCT | Ala | T | GTT | Val |
| *Rv2484c* | 1397 | G | GGT | Gly | A | GAT | Asp |
| *Rv2502c* | 973 | T | TTT | Phe | G | GTT | Val |
| *Rv2513* | 365 | C | ACG | Thr | A | AAG | Lys |
| *Rv2564* | 727 | A | ATG | Met | C | CTG | Leu |
| *Rv2688c* | 466 | C | CCC | Pro | A | ACC | Thr |
| *Rv2693c* | 376 | G | GGG | Gly | A | AGG | Arg |
| *Rv2696c* | 595 | C | CGG | Arg | G | GGG | Gly |
| *Rv2714* | 734 | T | GTG | Val | C | GCG | Ala |
| *Rv2719c* | 370 | T | TAT | Tyr | C | CAT | His |
| *Rv2729c* | 605 | C | GCG | Ala | A | GAG | Glu |
| *Rv2736c* | 175 | G | GTT | Val | C | CTT | Leu |
| *Rv2794c* | 259 | A | ATG | Met | G | GTG | Val |
| *Rv2800* | 202 | A | ACC | Thr | C | CCC | Pro |
| *Rv2869c* | 775 | G | GTC | Val | T | TTC | Phe |
| *Rv2905* | 242 | A | CAG | Gln | G | CGG | Arg |
| *Rv2941* | 1306 | A | ACA | Thr | G | GCA | Ala |
| *Rv3204* | 100 | A | ACA | Thr | G | GCA | Ala |
| *Rv3220c* | 286 | G | GGC | Gly | C | CGC | Arg |
| *Rv3331* | 1268 | C | CCG | Pro | T | CTG | Leu |
| *Rv3365c* | 2061 | T | AGT | Ser | G | AGG | Arg |
| *Rv3365c* | 2093 | A | CAG | Gln | G | CGG | Arg |
| *Rv3465* | 345 | A | GAA | Glu | C | GAC | Asp |
| *Rv3490* | 230 | A | GAA | Glu | G | GGA | Gly |
| *Rv3490* | 1000 | G | GTG | Val | C | CTG | Leu |
| *Rv3722c* | 473 | T | ATG | Met | C | ACG | Thr |
| *Rv3826* | 1264 | G | GAA | Glu | C | CAA | Gln |
| *Rv3837c* | 44 | G | GGC | Gly | A | GAC | Asp |
| *Rv3892c* | 56 | C | ACG | Thr | A | AAG | Lys |
| **wh18** | | | | | | | |
| *Rv0012* | 697 | T | TGC | Cys | C | CGC | Arg |
| *Rv0174* | 1109 | T | CTG | Leu | C | CCG | Pro |
| *Rv0192* | 379 | T | TCG | Ser | C | CCG | Pro |
| *Rv0221* | 721 | G | GCC | Ala | C | CCC | Pro |
| *Rv0284* | 641 | C | CCG | Pro | G | CGG | Arg |
| *Rv0647c* | 1066 | A | ACC | Thr | G | GCC | Ala |
| *Rv0667* | 1349 | C | TCG | Ser | T | TTG | Leu |
| *Rv0667* | 2917 | G | GGC | Gly | A | AGC | Ser |
| *Rv0787* | 799 | T | TAC | Tyr | C | CAC | His |
| *Rv0825c* | 134 | G | CGC | Arg | A | CAC | His |
| *Rv1286* | 572 | C | TCC | Ser | T | TTC | Phe |
| *Rv1486c* | 594 | G | AAG | Lys | T | AAT | Asn |
| *Rv1502* | 638 | A | TAC | Tyr | G | TGC | Cys |
| *Rv1569* | 598 | G | GCG | Ala | A | ACG | Thr |
| *Rv1815* | 247 | A | ATC | Ile | T | TTC | Phe |
| *Rv1848* | 21 | A | GAA | Glu | C | GAC | Asp |
| *Rv1860* | 406 | T | TTC | Phe | C | CTC | Leu |
| *Rv1900c* | 612 | T | ATT | Ile | G | ATG | Met |
| *Rv2003c* | 598 | T | TAC | Tyr | G | GAC | Asp |
| *Rv2090* | 1074 | C | TTC | Phe | G | TTG | Leu |
| *Rv2190c* | 259 | A | ACG | Thr | G | GCG | Ala |
| *Rv2406c* | 22 | C | CGG | Arg | G | GGG | Gly |
| *Rv2482c* | 2332 | T | TGC | Cys | C | CGC | Arg |
| *Rv2513* | 365 | C | ACG | Thr | A | AAG | Lys |
| *Rv2616* | 13 | G | GCG | Ala | A | ACG | Thr |
| *Rv2688c* | 466 | C | CCC | Pro | A | ACC | Thr |
| *Rv2729c* | 605 | C | GCG | Ala | A | GAG | Glu |
| *Rv2794c* | 259 | A | ATG | Met | G | GTG | Val |
| *Rv3204* | 100 | A | ACA | Thr | G | GCA | Ala |
| *Rv3331* | 1268 | C | CCG | Pro | T | CTG | Leu |
| *Rv3395c* | 134 | C | GCG | Ala | T | GTG | Val |
| *Rv3404c* | 195 | G | CAG | Gln | T | CAT | His |
| **sz9610** | | | | | | | |
| *Rv0012* | 697 | T | TGC | Cys | C | CGC | Arg |
| *Rv0012* | 773 | G | GGC | Gly | T | GTC | Val |
| *Rv0013* | 204 | C | ATC | Ile | G | ATG | Met |
| *Rv0169* | 187 | A | AAG | Lys | G | GAG | Glu |
| *Rv0169* | 937 | T | TCC | Ser | G | GCC | Ala |
| *Rv0172* | 563 | T | ATC | Ile | C | ACC | Thr |
| *Rv0174* | 1109 | T | CTG | Leu | C | CCG | Pro |
| *Rv0192* | 379 | T | TCG | Ser | C | CCG | Pro |
| *Rv0221* | 63 | G | ATG | Met | A | ATA | Ile |
| *Rv0252* | 2323 | G | GTG | Val | T | TTG | Leu |
| *Rv0284* | 641 | C | CCG | Pro | G | CGG | Arg |
| *Rv0290* | 227 | G | AGC | Ser | A | AAC | Asn |
| *Rv0290* | 283 | G | GCC | Ala | A | ACC | Thr |
| *Rv0398c* | 87 | A | GAA | Glu | C | GAC | Asp |
| *Rv0515* | 958 | C | CCG | Pro | T | TCG | Ser |
| *Rv0515* | 1127 | A | TAC | Tyr | G | TGC | Cys |
| *Rv0530* | 692 | C | CCC | Pro | T | CTC | Leu |
| *Rv0576* | 698 | G | CGC | Arg | A | CAC | His |
| *Rv0667* | 1333 | C | CAC | His | T | TAC | Tyr |
| *Rv0667* | 1361 | C | CCC | Pro | A | CAC | His |
| *Rv0697* | 1423 | G | GCC | Ala | C | CCC | Pro |
| *Rv0740* | 451 | C | CCG | Pro | A | ACG | Thr |
| *Rv0740* | 471 | A | CAA | Gln | G | GAG | Glu |
| *Rv0740* | 494 | G | GGG | Gly | T | GTG | Val |
| *Rv0740* | 499 | T | TCG | Ser | C | CCG | Pro |
| *Rv0740* | 517 | G | GCC | Ala | T | TCT | Ser |
| *Rv0787* | 799 | T | TAC | Tyr | C | CAC | His |
| *Rv0825c* | 203 | T | TTC | Phe | G | TGC | Cys |
| *Rv0844c* | 505 | G | GGT | Gly | C | CGT | Arg |
| *Rv1168c* | 500 | C | CCG | Pro | T | CTG | Leu |
| *Rv1170* | 445 | C | CAC | His | T | TAC | Tyr |
| *Rv1317c* | 34 | A | ATC | Ile | G | GTC | Val |
| *Rv1483* | 251 | T | GTG | Val | G | GGG | Gly |
| *Rv1486c* | 594 | G | AAG | Lys | T | AAT | Asn |
| *Rv1502* | 638 | A | TAC | Tyr | G | TGC | Cys |
| *Rv1592c* | 964 | A | ATT | Ile | G | GTT | Val |
| *Rv1647* | 4 | G | GCG | Ala | C | CCG | Pro |
| *Rv1815* | 247 | A | ATC | Ile | T | TTC | Phe |
| *Rv1821* | 1228 | G | GTG | Val | A | ATG | Met |
| *Rv1821* | 2220 | G | ATG | Met | T | ATT | Ile |
| *Rv1847* | 394 | C | CGG | Arg | T | TGG | Trp |
| *Rv1860* | 406 | T | TTC | Phe | C | CTC | Leu |
| *Rv1900c* | 612 | T | ATT | Ile | G | ATG | Met |
| *Rv2015c* | 453 | A | GAA | Glu | T | GAT | Asp |
| *Rv2057c* | 137 | G | CGT | Arg | T | CTT | Leu |
| *Rv2059* | 213 | C | CAC | His | A | CAA | Gln |
| *Rv2059* | 950 | C | ACA | Thr | A | AAA | Lys |
| *Rv2090* | 1074 | C | TTC | Phe | G | TTG | Leu |
| *Rv2351c* | 1336 | A | ACT | Thr | G | GCT | Ala |
| *Rv2409c* | 671 | C | ACC | Thr | A | AAC | Asn |
| *Rv2482c* | 2332 | T | TGC | Cys | C | CGC | Arg |
| *Rv2484c* | 1397 | G | GGT | Gly | A | GAT | Asp |
| *Rv2502c* | 973 | T | TTT | Phe | G | GTT | Val |
| *Rv2564* | 727 | A | ATG | Met | C | CTG | Leu |
| *Rv2688c* | 466 | C | CCC | Pro | A | ACC | Thr |
| *Rv2693c* | 376 | G | GGG | Gly | A | AGG | Arg |
| *Rv2714* | 734 | T | GTG | Val | C | GCG | Ala |
| *Rv2719c* | 370 | T | TAT | Tyr | C | CAT | His |
| *Rv2729c* | 605 | C | GCG | Ala | A | GAG | Glu |
| *Rv2736c* | 175 | G | GTT | Val | C | CTT | Leu |
| *Rv2736c* | 371 | T | CTG | Leu | G | CGG | Arg |
| *Rv2794c* | 259 | A | ATG | Met | G | GTG | Val |
| *Rv2869c* | 775 | G | GTC | Val | T | TTC | Phe |
| *Rv2905* | 242 | A | CAG | Gln | G | CGG | Arg |
| *Rv2941* | 1306 | A | ACA | Thr | G | GCA | Ala |
| *Rv3204* | 100 | A | ACA | Thr | G | GCA | Ala |
| *Rv3220c* | 286 | G | GGC | Gly | C | CGC | Arg |
| *Rv3331* | 1268 | C | CCG | Pro | T | CTG | Leu |
| *Rv3365c* | 2061 | T | AGT | Ser | G | AGG | Arg |
| *Rv3365c* | 2093 | A | CAG | Gln | G | CGG | Arg |
| *Rv3465* | 345 | A | GAA | Glu | C | GAC | Asp |
| *Rv3490* | 230 | A | GAA | Glu | G | GGA | Gly |
| *Rv3490* | 1000 | G | GTG | Val | C | CTG | Leu |
| *Rv3490* | 1081 | C | CAC | His | T | TAC | Tyr |
| *Rv3587c* | 730 | G | GGG | Gly | C | CGG | Arg |
| *Rv3722c* | 473 | T | ATG | Met | C | ACG | Thr |
| *Rv3826* | 1264 | G | GAA | Glu | C | CAA | Gln |
| *Rv3837c* | 44 | G | GGC | Gly | A | GAC | Asp |
| *Rv3892c* | 56 | C | ACG | Thr | A | AAG | Lys |
| **sz596** | | | | | | | |
| *Rv0012* | 697 | T | TGC | Cys | C | CGC | Arg |
| *Rv0012* | 773 | G | GGC | Gly | T | GTC | Val |
| *Rv0013* | 204 | C | ATC | Ile | G | ATG | Met |
| *Rv0169* | 937 | T | TCC | Ser | G | GCC | Ala |
| *Rv0172* | 563 | T | ATC | Ile | C | ACC | Thr |
| *Rv0174* | 1109 | T | CTG | Leu | C | CCG | Pro |
| *Rv0192* | 379 | T | TCG | Ser | C | CCG | Pro |
| *Rv0221* | 63 | G | ATG | Met | A | ATA | Ile |
| *Rv0252* | 2323 | G | GTG | Val | T | TTG | Leu |
| *Rv0284* | 641 | C | CCG | Pro | G | CGG | Arg |
| *Rv0290* | 227 | G | AGC | Ser | A | AAC | Asn |
| *Rv0290* | 283 | G | GCC | Ala | A | ACC | Thr |
| *Rv0398c* | 87 | A | GAA | Glu | C | GAC | Asp |
| *Rv0530* | 692 | C | CCC | Pro | T | CTC | Leu |
| *Rv0576* | 698 | G | CGC | Arg | A | CAC | His |
| *Rv0667* | 1349 | C | TCG | Ser | T | TTG | Leu |
| *Rv0697* | 1423 | G | GCC | Ala | C | CCC | Pro |
| *Rv0787* | 799 | T | TAC | Tyr | C | CAC | His |
| *Rv0825c* | 203 | T | TTC | Phe | G | TGC | Cys |
| *Rv0844c* | 505 | G | GGT | Gly | C | CGT | Arg |
| *Rv1168c* | 500 | C | CCG | Pro | T | CTG | Leu |
| *Rv1224* | 22 | T | TGG | Trp | G | GGG | Gly |
| *Rv1317c* | 34 | A | ATC | Ile | G | GTC | Val |
| *Rv1486c* | 594 | G | AAG | Lys | T | AAT | Asn |
| *Rv1502* | 638 | A | TAC | Tyr | G | TGC | Cys |
| *Rv1592c* | 964 | A | ATT | Ile | G | GTT | Val |
| *Rv1815* | 247 | A | ATC | Ile | T | TTC | Phe |
| *Rv1821* | 1228 | G | GTG | Val | A | ATG | Met |
| *Rv1847* | 394 | C | CGG | Arg | T | TGG | Trp |
| *Rv1860* | 406 | T | TTC | Phe | C | CTC | Leu |
| *Rv1900c* | 612 | T | ATT | Ile | G | ATG | Met |
| *Rv2015c* | 453 | A | GAA | Glu | T | GAT | Asp |
| *Rv2057c* | 137 | G | CGT | Arg | T | CTT | Leu |
| *Rv2059* | 213 | C | CAC | His | A | CAA | Gln |
| *Rv2059* | 950 | C | ACA | Thr | A | AAA | Lys |
| *Rv2090* | 1074 | C | TTC | Phe | G | TTG | Leu |
| *Rv2124c* | 3322 | A | AAG | Lys | C | CAG | Gln |
| *Rv2351c* | 1336 | A | ACT | Thr | G | GCT | Ala |
| *Rv2409c* | 671 | C | ACC | Thr | A | AAC | Asn |
| *Rv2482c* | 2332 | T | TGC | Cys | C | CGC | Arg |
| *Rv2484c* | 1397 | G | GGT | Gly | A | GAT | Asp |
| *Rv2502c* | 973 | T | TTT | Phe | G | GTT | Val |
| *Rv2513* | 365 | C | ACG | Thr | A | AAG | Lys |
| *Rv2564* | 727 | A | ATG | Met | C | CTG | Leu |
| *Rv2688c* | 466 | C | CCC | Pro | A | ACC | Thr |
| *Rv2693c* | 376 | G | GGG | Gly | A | AGG | Arg |
| *Rv2714* | 734 | T | GTG | Val | C | GCG | Ala |
| *Rv2719c* | 370 | T | TAT | Tyr | C | CAT | His |
| *Rv2729c* | 605 | C | GCG | Ala | A | GAG | Glu |
| *Rv2736c* | 175 | G | GTT | Val | C | CTT | Leu |
| *Rv2794c* | 259 | A | ATG | Met | G | GTG | Val |
| *Rv2869c* | 775 | G | GTC | Val | T | TTC | Phe |
| *Rv2905* | 242 | A | CAG | Gln | G | CGG | Arg |
| *Rv2941* | 1306 | A | ACA | Thr | G | GCA | Ala |
| *Rv3204* | 100 | A | ACA | Thr | G | GCA | Ala |
| *Rv3220c* | 286 | G | GGC | Gly | C | CGC | Arg |
| *Rv3331* | 1268 | C | CCG | Pro | T | CTG | Leu |
| *Rv3365c* | 2061 | T | AGT | Ser | G | AGG | Arg |
| *Rv3365c* | 2093 | A | CAG | Gln | G | CGG | Arg |
| *Rv3465* | 345 | A | GAA | Glu | C | GAC | Asp |
| *Rv3490* | 230 | A | GAA | Glu | G | GGA | Gly |
| *Rv3490* | 1000 | G | GTG | Val | C | CTG | Leu |
| *Rv3722c* | 473 | T | ATG | Met | C | ACG | Thr |
| *Rv3826* | 1264 | G | GAA | Glu | C | CAA | Gln |
| *Rv3837c* | 44 | G | GGC | Gly | A | GAC | Asp |
| *Rv3892c* | 56 | C | ACG | Thr | A | AAG | Lys |
| **sz6213** | | | | | | | |
| *Rv0012* | 697 | T | TGC | Cys | C | CGC | Arg |
| *Rv0012* | 773 | G | GGC | Gly | T | GTC | Val |
| *Rv0013* | 204 | C | ATC | Ile | G | ATG | Met |
| *Rv0169* | 937 | T | TCC | Ser | G | GCC | Ala |
| *Rv0172* | 563 | T | ATC | Ile | C | ACC | Thr |
| *Rv0174* | 1109 | T | CTG | Leu | C | CCG | Pro |
| *Rv0192* | 379 | T | TCG | Ser | C | CCG | Pro |
| *Rv0221* | 63 | G | ATG | Met | A | ATA | Ile |
| *Rv0252* | 2323 | G | GTG | Val | T | TTG | Leu |
| *Rv0284* | 641 | C | CCG | Pro | G | CGG | Arg |
| *Rv0290* | 227 | G | AGC | Ser | A | AAC | Asn |
| *Rv0290* | 283 | G | GCC | Ala | A | ACC | Thr |
| *Rv0398c* | 87 | A | GAA | Glu | C | GAC | Asp |
| *Rv0530* | 692 | C | CCC | Pro | T | CTC | Leu |
| *Rv0576* | 698 | G | CGC | Arg | A | CAC | His |
| *Rv0667* | 1349 | C | TCG | Ser | T | TTG | Leu |
| *Rv0697* | 1423 | G | GCC | Ala | C | CCC | Pro |
| *Rv0787* | 799 | T | TAC | Tyr | C | CAC | His |
| *Rv0825c* | 203 | T | TTC | Phe | G | TGC | Cys |
| *Rv0844c* | 505 | G | GGT | Gly | C | CGT | Arg |
| *Rv1168c* | 500 | C | CCG | Pro | T | CTG | Leu |
| *Rv1224* | 22 | T | TGG | Trp | G | GGG | Gly |
| *Rv1316c* | 286 | G | GGG | Gly | C | CGG | Arg |
| *Rv1317c* | 34 | A | ATC | Ile | G | GTC | Val |
| *Rv1486c* | 594 | G | AAG | Lys | T | AAT | Asn |
| *Rv1502* | 638 | A | TAC | Tyr | G | TGC | Cys |
| *Rv1592c* | 964 | A | ATT | Ile | G | GTT | Val |
| *Rv1815* | 247 | A | ATC | Ile | T | TTC | Phe |
| *Rv1821* | 1228 | G | GTG | Val | A | ATG | Met |
| *Rv1847* | 394 | C | CGG | Arg | T | TGG | Trp |
| *Rv1860* | 406 | T | TTC | Phe | C | CTC | Leu |
| *Rv1900c* | 612 | T | ATT | Ile | G | ATG | Met |
| *Rv2015c* | 453 | A | GAA | Glu | T | GAT | Asp |
| *Rv2057c* | 137 | G | CGT | Arg | T | CTT | Leu |
| *Rv2059* | 213 | C | CAC | His | A | CAA | Gln |
| *Rv2059* | 950 | C | ACA | Thr | A | AAA | Lys |
| *Rv2064* | 808 | A | ATC | Ile | T | TTC | Phe |
| *Rv2090* | 1074 | C | TTC | Phe | G | TTG | Leu |
| *Rv2124c* | 2156 | A | CAC | His | G | CGC | Arg |
| *Rv2351c* | 1336 | A | ACT | Thr | G | GCT | Ala |
| *Rv2409c* | 671 | C | ACC | Thr | A | AAC | Asn |
| *Rv2482c* | 2332 | T | TGC | Cys | C | CGC | Arg |
| *Rv2484c* | 1397 | G | GGT | Gly | A | GAT | Asp |
| *Rv2502c* | 973 | T | TTT | Phe | G | GTT | Val |
| *Rv2513* | 365 | C | ACG | Thr | A | AAG | Lys |
| *Rv2564* | 727 | A | ATG | Met | C | CTG | Leu |
| *Rv2688c* | 466 | C | CCC | Pro | A | ACC | Thr |
| *Rv2693c* | 376 | G | GGG | Gly | A | AGG | Arg |
| *Rv2714* | 734 | T | GTG | Val | C | GCG | Ala |
| *Rv2719c* | 370 | T | TAT | Tyr | C | CAT | His |
| *Rv2729c* | 605 | C | GCG | Ala | A | GAG | Glu |
| *Rv2736c* | 175 | G | GTT | Val | C | CTT | Leu |
| *Rv2794c* | 259 | A | ATG | Met | G | GTG | Val |
| *Rv2869c* | 775 | G | GTC | Val | T | TTC | Phe |
| *Rv2905* | 242 | A | CAG | Gln | G | CGG | Arg |
| *Rv2941* | 1306 | A | ACA | Thr | G | GCA | Ala |
| *Rv3204* | 100 | A | ACA | Thr | G | GCA | Ala |
| *Rv3220c* | 286 | G | GGC | Gly | C | CGC | Arg |
| *Rv3331* | 1268 | C | CCG | Pro | T | CTG | Leu |
| *Rv3465* | 345 | A | GAA | Glu | C | GAC | Asp |
| *Rv3490* | 230 | A | GAA | Glu | G | GGA | Gly |
| *Rv3490* | 1000 | G | GTG | Val | C | CTG | Leu |
| *Rv3722c* | 473 | T | ATG | Met | C | ACG | Thr |
| *Rv3826* | 1264 | G | GAA | Glu | C | CAA | Gln |
| *Rv3837c* | 44 | G | GGC | Gly | A | GAC | Asp |
| *Rv3879c* | 1979 | A | GAG | Glu | C | GCG | Ala |
| *Rv3879c* | 2014 | C | CGC | Arg | G | GGC | Gly |
| *Rv3879c* | 2186 | G | TGC | Cys | C | TCC | Ser |
| *Rv3892c* | 56 | C | ACG | Thr | A | AAG | Lys |
| **wx6** | | | | | | | |
| *Rv0012* | 697 | T | TGC | Cys | C | CGC | Arg |
| *Rv0106* | 1187 | A | GAA | Glu | G | GGA | Gly |
| *Rv0174* | 1109 | T | CTG | Leu | C | CCG | Pro |
| *Rv0192* | 379 | T | TCG | Ser | C | CCG | Pro |
| *Rv0284* | 641 | C | CCG | Pro | G | CGG | Arg |
| *Rv0284* | 3566 | G | CGC | Arg | A | CAC | His |
| *Rv0530* | 797 | G | GGC | Gly | A | GAC | Asp |
| *Rv0571c* | 1016 | C | GCG | Ala | T | GTG | Val |
| *Rv0787* | 799 | T | TAC | Tyr | C | CAC | His |
| *Rv1010* | 775 | G | GGA | Gly | C | CGA | Arg |
| *Rv1486c* | 594 | G | AAG | Lys | T | AAT | Asn |
| *Rv1587c* | 962 | G | CGA | Arg | C | CCA | Pro |
| *Rv1587c* | 982 | C | CAC | His | A | AAC | Asn |
| *Rv1587c* | 998 | G | CGT | Arg | C | CCT | Pro |
| *Rv1637c* | 16 | A | ACC | Thr | G | GCC | Ala |
| *Rv1637c* | 19 | G | GAC | Asp | A | AAC | Asn |
| *Rv1637c* | 22 | A | AAC | Asn | G | GAC | Asp |
| *Rv1637c* | 25 | C | CAT | His | G | GAT | Asp |
| *Rv1637c* | 28 | C | CAG | Gln | G | GAT | Asp |
| *Rv1637c* | 45 | C | GTC | Val | A | ATA | Ile |
| *Rv1637c* | 47 | T | GTA | Val | G | GGA | Gly |
| *Rv1637c* | 49 | A | ACG | Thr | G | GCA | Ala |
| *Rv1663* | 179 | C | GCG | Ala | G | GGG | Gly |
| *Rv1788* | 143 | C | TCG | Ser | G | TGG | Trp |
| *Rv1815* | 247 | A | ATC | Ile | T | TTC | Phe |
| *Rv1860* | 406 | T | TTC | Phe | C | CTC | Leu |
| *Rv1900c* | 612 | T | ATT | Ile | G | ATG | Met |
| *Rv1911c* | 325 | T | TAC | Tyr | C | CAC | His |
| *Rv2003c* | 440 | C | CCT | Pro | T | CTT | Leu |
| *Rv2220* | 1315 | G | GAA | Glu | A | AAA | Lys |
| *Rv2482c* | 2332 | T | TGC | Cys | C | CGC | Arg |
| *Rv2503c* | 578 | C | ACC | Thr | T | ATC | Ile |
| *Rv2513* | 365 | C | ACG | Thr | A | AAG | Lys |
| *Rv2688c* | 466 | C | CCC | Pro | A | ACC | Thr |
| *Rv2729c* | 605 | C | GCG | Ala | A | GAG | Glu |
| *Rv2794c* | 259 | A | ATG | Met | G | GTG | Val |
| *Rv2869c* | 586 | C | CCG | Pro | T | TCG | Ser |
| *Rv3204* | 100 | A | ACA | Thr | G | GCA | Ala |
| *Rv3331* | 1268 | C | CCG | Pro | T | CTG | Leu |
| *Rv3709c* | 49 | G | GAA | Glu | A | AAA | Lys |
| *Rv3863* | 895 | G | GCC | Ala | A | ACC | Thr |
| *Rv3892c* | 56 | C | ACG | Thr | A | AAG | Lys |

Table S8 Number of dysregulated genes in each rifampicin-resistant isolate toward rifampicin exposure.

| **Isolates** | **Number of genes** | | | **Proportion of dysregulated genes** |
| --- | --- | --- | --- | --- |
|  | **Upregulated** | **Downregulated** | **Not differentially expressed** |  |
| wx2 | 356 | 380 | 3033 | 19.53% |
| wh18 | 2905 | 560 | 304 | 91.93% |
| sz9610 | 1060 | 501 | 2208 | 41.42% |
| sz596 | 1519 | 431 | 1819 | 51.74% |
| sz6213 | 1977 | 535 | 1257 | 66.65% |
| wx6 | 181 | 926 | 2662 | 29.37% |

Table S10 Summary of SNPs in dysregulated genes associated with *rpoC* mutations in *M. tuberculosis* rifampin-resistant isolates following rifampin exposure.

| **Synonym** | **Location** | **Reference** | | | **Open reading frame** | | | |
| --- | --- | --- | --- | --- | --- | --- | --- | --- |
|  |  | **Base** | **Codon** | **Amino acid** | **Base** | **Codon** | **Amino acid** | |
| **wx2** | | | | | | | |  |
| *Rv0010c* | 266 | T | ATC | Ile | A | AAC | Asn | |
| *Rv0015c* | 1106 | A | CAG | Gln | G | CGG | Arg | |
| *Rv0015c* | 1109 | A | CAG | Gln | C | CCG | Pro | |
| *Rv0018c* | 1387 | C | CCG | Pro | T | TCG | Ser | |
| *Rv0039c* | 71 | G | TGC | Cys | T | TTC | Phe | |
| *Rv0078A* | 334 | G | GAG | Glu | A | AAG | Lys | |
| *Rv0368c* | 746 | G | CGT | Arg | A | CAT | His | |
| *Rv0426c* | 290 | C | CCC | Pro | T | CTC | Leu | |
| *Rv0444c* | 243 | G | GAG | Glu | C | GAC | Asp | |
| *Rv0452* | 373 | C | CAT | His | G | GAT | Asp | |
| *Rv0537c* | 868 | A | ACC | Thr | G | GCC | Ala | |
| *Rv0545c* | 145 | C | CCT | Pro | T | TCT | Ser | |
| *Rv0556* | 44 | T | CTC | Leu | G | CGC | Arg | |
| *Rv0565c* | 329 | G | CGC | Arg | A | CAC | His | |
| *Rv0629c* | 360 | A | GAA | Glu | C | GAC | Asp | |
| *Rv0679c* | 426 | C | AAC | Asn | G | AAG | Lys | |
| *Rv0728c* | 725 | G | CGC | Arg | A | CAC | His | |
| *Rv0747* | 2344 | A | AGC | Ser | G | GCC | Ala | |
| *Rv0747* | 2345 | G | AGC | Ser | C | GCC | Ala | |
| *Rv0758* | 515 | C | CCC | Pro | T | CTC | Leu | |
| *Rv0859* | 448 | A | AGC | Ser | G | GGC | Gly | |
| *Rv0922* | 533 | G | GGC | Gly | A | GAC | Asp | |
| *Rv0982* | 1016 | T | CTT | Leu | A | CAT | His | |
| *Rv0989c* | 961 | A | ATA | Ile | G | GTA | Val | |
| *Rv0995* | 67 | G | GGC | Gly | A | AGC | Ser | |
| *Rv1102c* | 194 | C | ACC | Thr | T | ATC | Ile | |
| *Rv1163* | 536 | C | CCG | Pro | G | CGG | Arg | |
| *Rv1239c* | 415 | A | AAA | Lys | G | GAA | Glu | |
| *Rv1248c* | 2290 | G | GTG | Val | A | ATG | Met | |
| *Rv1266c* | 1820 | G | CGG | Arg | A | CAG | Gln | |
| *Rv1446c* | 575 | G | CGA | Arg | C | CCA | Pro | |
| *Rv1449c* | 52 | T | TAC | Tyr | G | GAC | Asp | |
| *Rv1538c* | 841 | G | GGC | Gly | A | AGC | Ser | |
| *Rv1597* | 62 | G | GGT | Gly | A | GAT | Asp | |
| *Rv1716* | 532 | A | AGT | Ser | G | GGT | Gly | |
| *Rv1716* | 827 | T | GTC | Val | C | GCC | Ala | |
| *Rv1733c* | 204 | G | CAG | Gln | T | CAT | His | |
| *Rv1802* | 512 | C | CCG | Pro | T | CTG | Leu | |
| *Rv1826* | 362 | C | ACA | Thr | A | AAA | Lys | |
| *Rv1837c* | 310 | G | GGT | Gly | A | AGT | Ser | |
| *Rv2109c* | 404 | G | CGC | Arg | C | CCC | Pro | |
| *Rv2109c* | 544 | C | CGT | Arg | G | GGT | Gly | |
| *Rv2127* | 26 | G | GGC | Gly | A | GAC | Asp | |
| *Rv2187* | 299 | C | ACC | Thr | T | ATC | Ile | |
| *Rv2383c* | 2020 | G | GTG | Val | C | CTG | Leu | |
| *Rv2416c* | 487 | G | GTC | Val | A | ATC | Ile | |
| *Rv2450c* | 59 | C | ACG | Thr | G | AGG | Arg | |
| *Rv2450c* | 377 | G | CGG | Arg | A | CAG | Gln | |
| *Rv2494* | 143 | T | GTG | Val | C | GCG | Ala | |
| *Rv2495c* | 307 | T | TAC | Tyr | G | GAC | Asp | |
| *Rv2495c* | 319 | A | ACC | Thr | G | GCC | Ala | |
| *Rv2524c* | 8311 | T | TGC | Cys | C | CGC | Arg | |
| *Rv2542* | 631 | A | ACC | Thr | G | GCC | Ala | |
| *Rv2685* | 1133 | C | GCA | Ala | G | GGA | Gly | |
| *Rv2715* | 787 | C | CCG | Pro | T | TCG | Ser | |
| *Rv2724c* | 466 | G | GTG | Val | A | ATG | Met | |
| *Rv3014c* | 1570 | C | CCG | Pro | A | ACG | Thr | |
| *Rv3014c* | 1573 | C | CTG | Leu | A | AAC | Asn | |
| *Rv3014c* | 1574 | T | CTG | Leu | A | AAC | Asn | |
| *Rv3014c* | 1624 | G | GCC | Ala | A | AAC | Asn | |
| *Rv3014c* | 1625 | C | GCC | Ala | A | AAC | Asn | |
| *Rv3014c* | 1627 | C | CGC | Arg | T | TGT | Cys | |
| *Rv3014c* | 1629 | C | CGC | Arg | T | TGT | Cys | |
| *Rv3014c* | 1652 | G | AGC | Ser | A | AAA | Lys | |
| *Rv3015c* | 500 | T | TTC | Phe | G | TGC | Cys | |
| *Rv3051c* | 1556 | A | CAG | Gln | G | CGG | Arg | |
| *Rv3113* | 401 | G | GGA | Gly | A | GAA | Glu | |
| *Rv3236c* | 304 | A | ACG | Thr | G | GCG | Ala | |
| *Rv3296* | 1475 | A | AAG | Lys | C | ACG | Thr | |
| *Rv3317* | 160 | G | GTG | Val | C | CTG | Leu | |
| *Rv3317* | 334 | A | ACC | Thr | G | GCC | Ala | |
| *Rv3411c* | 1171 | G | GCG | Ala | A | ACG | Thr | |
| *Rv3586* | 862 | A | ACG | Thr | G | GCG | Ala | |
| *Rv3706c* | 127 | T | TTC | Phe | G | GTC | Val | |
| *Rv3715c* | 130 | G | GGT | Gly | T | TGT | Cys | |
| *Rv3729* | 805 | C | CCG | Pro | T | TCG | Ser | |
| *Rv3729* | 1548 | G | CAG | Gln | C | CAC | His | |
| *Rv3777* | 479 | T | GTC | Val | C | GCC | Ala | |
| *Rv3800c* | 3041 | C | ACG | Thr | T | ATG | Met | |
| *Rv3800c* | 4937 | C | GCC | Ala | A | GAC | Asp | |
| **wh18** | | | | | | | |  |
| *Rv0018c* | 1387 | C | CCG | Pro | T | TCG | Ser | |
| *Rv0248c* | 611 | C | GCG | Ala | T | GTG | Val | |
| *Rv0368c* | 89 | T | GTG | Val | C | GCG | Ala | |
| *Rv0512* | 560 | A | GAC | Asp | C | GCC | Ala | |
| *Rv0545c* | 145 | C | CCT | Pro | T | TCT | Ser | |
| *Rv0650* | 133 | G | GTC | Val | A | ATC | Ile | |
| *Rv0758* | 515 | C | CCC | Pro | T | CTC | Leu | |
| *Rv0859* | 448 | A | AGC | Ser | G | GGC | Gly | |
| *Rv0989c* | 961 | A | ATA | Ile | G | GTA | Val | |
| *Rv1003* | 94 | G | GTG | Val | A | ATG | Met | |
| *Rv1182* | 641 | G | CGC | Arg | A | CAC | His | |
| *Rv1239c* | 415 | A | AAA | Lys | G | GAA | Glu | |
| *Rv1266c* | 1820 | G | CGG | Arg | A | CAG | Gln | |
| *Rv1309* | 473 | T | GTG | Val | C | GCG | Ala | |
| *Rv1371* | 1390 | C | CGC | Arg | T | TGC | Cys | |
| *Rv1446c* | 575 | G | CGA | Arg | C | CCA | Pro | |
| *Rv1449c* | 52 | T | TAC | Tyr | G | GAC | Asp | |
| *Rv1480* | 848 | G | GGT | Gly | C | GCT | Ala | |
| *Rv1597* | 62 | G | GGT | Gly | A | GAT | Asp | |
| *Rv1716* | 532 | A | AGT | Ser | G | GGT | Gly | |
| *Rv1716* | 827 | T | GTC | Val | C | GCC | Ala | |
| *Rv1733c* | 204 | G | CAG | Gln | T | CAT | His | |
| *Rv1802* | 1223 | T | GTG | Val | G | GGG | Gly | |
| *Rv1845c* | 640 | C | CGC | Arg | T | TGC | Cys | |
| *Rv2164c* | 768 | T | GAT | Asp | G | GAG | Glu | |
| *Rv2450c* | 377 | G | CGG | Arg | A | CAG | Gln | |
| *Rv2495c* | 319 | A | ACC | Thr | G | GCC | Ala | |
| *Rv2542* | 631 | A | ACC | Thr | G | GCC | Ala | |
| *Rv2724c* | 855 | C | TTC | Phe | A | TTA | Leu | |
| *Rv3050c* | 422 | T | GTA | Val | C | GCA | Ala | |
| *Rv3058c* | 28 | C | CAG | Gln | G | GAG | Glu | |
| *Rv3104c* | 548 | G | GGT | Gly | T | GTT | Val | |
| *Rv3206c* | 71 | G | AGC | Ser | A | AAC | Asn | |
| *Rv3251c* | 104 | A | GAC | Asp | G | GGC | Gly | |
| *Rv3317* | 160 | G | GTG | Val | C | CTG | Leu | |
| *Rv3317* | 250 | T | TGG | Trp | C | CGG | Arg | |
| *Rv3677c* | 439 | G | GAC | Asp | A | AAC | Asn | |
| **sz9610** | | | | | | | |  |
| *Rv0015c* | 1106 | A | CAG | Gln | G | CGG | Arg | |
| *Rv0015c* | 1109 | A | CAG | Gln | C | CCG | Pro | |
| *Rv0018c* | 1387 | C | CCG | Pro | T | TCG | Ser | |
| *Rv0039c* | 71 | G | TGC | Cys | T | TTC | Phe | |
| *Rv0078A* | 334 | G | GAG | Glu | A | AAG | Lys | |
| *Rv0201c* | 8 | T | TTG | Leu | C | TCG | Ser | |
| *Rv0201c* | 10 | G | GCA | Ala | A | ACC | Thr | |
| *Rv0201c* | 12 | A | GCA | Ala | C | ACC | Thr | |
| *Rv0201c* | 14 | C | GCC | Ala | G | GGC | Gly | |
| *Rv0201c* | 17 | A | GAG | Glu | G | GGG | Gly | |
| *Rv0201c* | 23 | A | CAC | His | C | CCC | Pro | |
| *Rv0336* | 958 | C | CCG | Pro | T | TCG | Ser | |
| *Rv0336* | 1127 | A | TAC | Tyr | G | TGC | Cys | |
| *Rv0368c* | 746 | G | CGT | Arg | A | CAT | His | |
| *Rv0375c* | 187 | C | CGG | Arg | T | TGG | Trp | |
| *Rv0444c* | 243 | G | GAG | Glu | C | GAC | Asp | |
| *Rv0452* | 373 | C | CAT | His | G | GAT | Asp | |
| *Rv0537c* | 868 | A | ACC | Thr | G | GCC | Ala | |
| *Rv0545c* | 145 | C | CCT | Pro | T | TCT | Ser | |
| *Rv0556* | 44 | T | CTC | Leu | G | CGC | Arg | |
| *Rv0565c* | 329 | G | CGC | Arg | A | CAC | His | |
| *Rv0568* | 899 | C | GCC | Ala | G | GGC | Gly | |
| *Rv0629c* | 360 | A | GAA | Glu | C | GAC | Asp | |
| *Rv0679c* | 426 | C | AAC | Asn | G | AAG | Lys | |
| *Rv0728c* | 725 | G | CGC | Arg | A | CAC | His | |
| *Rv0758* | 515 | C | CCC | Pro | T | CTC | Leu | |
| *Rv0859* | 448 | A | AGC | Ser | G | GGC | Gly | |
| *Rv0922* | 533 | G | GGC | Gly | A | GAC | Asp | |
| *Rv0982* | 1016 | T | CTT | Leu | A | CAT | His | |
| *Rv0988* | 571 | C | CTG | Leu | G | GCG | Ala | |
| *Rv0988* | 572 | T | CTG | Leu | C | GCG | Ala | |
| *Rv0989c* | 961 | A | ATA | Ile | G | GTA | Val | |
| *Rv0995* | 67 | G | GGC | Gly | A | AGC | Ser | |
| *Rv1102c* | 194 | C | ACC | Thr | T | ATC | Ile | |
| *Rv1163* | 536 | C | CCG | Pro | G | CGG | Arg | |
| *Rv1239c* | 415 | A | AAA | Lys | G | GAA | Glu | |
| *Rv1248c* | 2290 | G | GTG | Val | A | ATG | Met | |
| *Rv1266c* | 1820 | G | CGG | Arg | A | CAG | Gln | |
| *Rv1425* | 382 | G | GGC | Gly | T | TGC | Cys | |
| *Rv1446c* | 575 | G | CGA | Arg | C | CCA | Pro | |
| *Rv1449c* | 52 | T | TAC | Tyr | G | GAC | Asp | |
| *Rv1538c* | 841 | G | GGC | Gly | A | AGC | Ser | |
| *Rv1597* | 62 | G | GGT | Gly | A | GAT | Asp | |
| *Rv1716* | 532 | A | AGT | Ser | G | GGT | Gly | |
| *Rv1716* | 827 | T | GTC | Val | C | GCC | Ala | |
| *Rv1733c* | 204 | G | CAG | Gln | T | CAT | His | |
| *Rv1826* | 362 | C | ACA | Thr | A | AAA | Lys | |
| *Rv1837c* | 310 | G | GGT | Gly | A | AGT | Ser | |
| *Rv2109c* | 404 | G | CGC | Arg | C | CCC | Pro | |
| *Rv2109c* | 544 | C | CGT | Arg | G | GGT | Gly | |
| *Rv2127* | 26 | G | GGC | Gly | A | GAC | Asp | |
| *Rv2136c* | 792 | G | ATG | Met | A | ATA | Ile | |
| *Rv2187* | 299 | C | ACC | Thr | T | ATC | Ile | |
| *Rv2196* | 1472 | A | AAG | Lys | C | ACG | Thr | |
| *Rv2383c* | 2020 | G | GTG | Val | C | CTG | Leu | |
| *Rv2450c* | 377 | G | CGG | Arg | A | CAG | Gln | |
| *Rv2494* | 143 | T | GTG | Val | C | GCG | Ala | |
| *Rv2495c* | 307 | T | TAC | Tyr | G | GAC | Asp | |
| *Rv2495c* | 319 | A | ACC | Thr | G | GCC | Ala | |
| *Rv2524c* | 8311 | T | TGC | Cys | C | CGC | Arg | |
| *Rv2524c* | 9053 | A | CAG | Gln | G | CGG | Arg | |
| *Rv2542* | 631 | A | ACC | Thr | G | GCC | Ala | |
| *Rv2685* | 1133 | C | GCA | Ala | G | GGA | Gly | |
| *Rv2724c* | 466 | G | GTG | Val | A | ATG | Met | |
| *Rv2931* | 3581 | T | CTA | Leu | G | CGA | Arg | |
| *Rv3015c* | 500 | T | TTC | Phe | G | TGC | Cys | |
| *Rv3015c* | 622 | G | GTC | Val | A | ATC | Ile | |
| *Rv3051c* | 1556 | A | CAG | Gln | G | CGG | Arg | |
| *Rv3113* | 401 | G | GGA | Gly | A | GAA | Glu | |
| *Rv3227* | 262 | G | GCT | Ala | A | ACT | Thr | |
| *Rv3236c* | 304 | A | ACG | Thr | G | GCG | Ala | |
| *Rv3296* | 1475 | A | AAG | Lys | C | ACG | Thr | |
| *Rv3317* | 160 | G | GTG | Val | C | CTG | Leu | |
| *Rv3317* | 334 | A | ACC | Thr | G | GCC | Ala | |
| *Rv3355c* | 176 | A | GAG | Glu | T | GTG | Val | |
| *Rv3411c* | 1171 | G | GCG | Ala | A | ACG | Thr | |
| *Rv3586* | 862 | A | ACG | Thr | G | GCG | Ala | |
| *Rv3715c* | 130 | G | GGT | Gly | T | TGT | Cys | |
| *Rv3729* | 805 | C | CCG | Pro | T | TCG | Ser | |
| *Rv3729* | 1548 | G | CAG | Gln | C | CAC | His | |
| *Rv3777* | 479 | T | GTC | Val | C | GCC | Ala | |
| *Rv3800c* | 3041 | C | ACG | Thr | T | ATG | Met | |
| *Rv3800c* | 4937 | C | GCC | Ala | A | GAC | Asp | |
| **sz596** | | | | | | | |  |
| *Rv0015c* | 1106 | A | CAG | Gln | G | CGG | Arg | |
| *Rv0015c* | 1109 | A | CAG | Gln | C | CCG | Pro | |
| *Rv0018c* | 1387 | C | CCG | Pro | T | TCG | Ser | |
| *Rv0039c* | 71 | G | TGC | Cys | T | TTC | Phe | |
| *Rv0078A* | 334 | G | GAG | Glu | A | AAG | Lys | |
| *Rv0154c* | 454 | G | GGT | Gly | T | TGT | Cys | |
| *Rv0368c* | 746 | G | CGT | Arg | A | CAT | His | |
| *Rv0444c* | 243 | G | GAG | Glu | C | GAC | Asp | |
| *Rv0452* | 373 | C | CAT | His | G | GAT | Asp | |
| *Rv0537c* | 868 | A | ACC | Thr | G | GCC | Ala | |
| *Rv0545c* | 145 | C | CCT | Pro | T | TCT | Ser | |
| *Rv0556* | 44 | T | CTC | Leu | G | CGC | Arg | |
| *Rv0565c* | 329 | G | CGC | Arg | A | CAC | His | |
| *Rv0629c* | 360 | A | GAA | Glu | C | GAC | Asp | |
| *Rv0679c* | 426 | C | AAC | Asn | G | AAG | Lys | |
| *Rv0728c* | 725 | G | CGC | Arg | A | CAC | His | |
| *Rv0747* | 2344 | A | AGC | Ser | G | GCC | Ala | |
| *Rv0747* | 2345 | G | AGC | Ser | C | GCC | Ala | |
| *Rv0758* | 515 | C | CCC | Pro | T | CTC | Leu | |
| *Rv0859* | 448 | A | AGC | Ser | G | GGC | Gly | |
| *Rv0922* | 533 | G | GGC | Gly | A | GAC | Asp | |
| *Rv0982* | 1016 | T | CTT | Leu | A | CAT | His | |
| *Rv0988* | 571 | C | CTG | Leu | G | GCG | Ala | |
| *Rv0988* | 572 | T | CTG | Leu | C | GCG | Ala | |
| *Rv0989c* | 961 | A | ATA | Ile | G | GTA | Val | |
| *Rv0995* | 67 | G | GGC | Gly | A | AGC | Ser | |
| *Rv1102c* | 194 | C | ACC | Thr | T | ATC | Ile | |
| *Rv1163* | 536 | C | CCG | Pro | G | CGG | Arg | |
| *Rv1239c* | 415 | A | AAA | Lys | G | GAA | Glu | |
| *Rv1248c* | 2290 | G | GTG | Val | A | ATG | Met | |
| *Rv1266c* | 1820 | G | CGG | Arg | A | CAG | Gln | |
| *Rv1446c* | 575 | G | CGA | Arg | C | CCA | Pro | |
| *Rv1449c* | 52 | T | TAC | Tyr | G | GAC | Asp | |
| *Rv1538c* | 841 | G | GGC | Gly | A | AGC | Ser | |
| *Rv1597* | 62 | G | GGT | Gly | A | GAT | Asp | |
| *Rv1615* | 239 | T | CTG | Leu | G | CGG | Arg | |
| *Rv1716* | 532 | A | AGT | Ser | G | GGT | Gly | |
| *Rv1716* | 827 | T | GTC | Val | C | GCC | Ala | |
| *Rv1733c* | 204 | G | CAG | Gln | T | CAT | His | |
| *Rv1826* | 362 | C | ACA | Thr | A | AAA | Lys | |
| *Rv1837c* | 310 | G | GGT | Gly | A | AGT | Ser | |
| *Rv2109c* | 404 | G | CGC | Arg | C | CCC | Pro | |
| *Rv2109c* | 544 | C | CGT | Arg | G | GGT | Gly | |
| *Rv2127* | 26 | G | GGC | Gly | A | GAC | Asp | |
| *Rv2187* | 299 | C | ACC | Thr | T | ATC | Ile | |
| *Rv2383c* | 2020 | G | GTG | Val | C | CTG | Leu | |
| *Rv2450c* | 377 | G | CGG | Arg | A | CAG | Gln | |
| *Rv2494* | 143 | T | GTG | Val | C | GCG | Ala | |
| *Rv2495c* | 307 | T | TAC | Tyr | G | GAC | Asp | |
| *Rv2495c* | 319 | A | ACC | Thr | G | GCC | Ala | |
| *Rv2524c* | 8311 | T | TGC | Cys | C | CGC | Arg | |
| *Rv2542* | 631 | A | ACC | Thr | G | GCC | Ala | |
| *Rv2685* | 1133 | C | GCA | Ala | G | GGA | Gly | |
| *Rv2724c* | 466 | G | GTG | Val | A | ATG | Met | |
| *Rv2837c* | 709 | G | GAG | Glu | A | AAG | Lys | |
| *Rv3015c* | 500 | T | TTC | Phe | G | TGC | Cys | |
| *Rv3051c* | 1556 | A | CAG | Gln | G | CGG | Arg | |
| *Rv3113* | 401 | G | GGA | Gly | A | GAA | Glu | |
| *Rv3236c* | 304 | A | ACG | Thr | G | GCG | Ala | |
| *Rv3296* | 1475 | A | AAG | Lys | C | ACG | Thr | |
| *Rv3296* | 2770 | G | GGG | Gly | C | CGG | Arg | |
| *Rv3317* | 160 | G | GTG | Val | C | CTG | Leu | |
| *Rv3317* | 334 | A | ACC | Thr | G | GCC | Ala | |
| *Rv3350c* | 8603 | C | GCC | Ala | T | GTC | Val | |
| *Rv3411c* | 1171 | G | GCG | Ala | A | ACG | Thr | |
| *Rv3586* | 862 | A | ACG | Thr | G | GCG | Ala | |
| *Rv3603c* | 112 | G | GTG | Val | A | ATG | Met | |
| *Rv3715c* | 130 | G | GGT | Gly | T | TGT | Cys | |
| *Rv3729* | 805 | C | CCG | Pro | T | TCG | Ser | |
| *Rv3729* | 1548 | G | CAG | Gln | C | CAC | His | |
| *Rv3777* | 479 | T | GTC | Val | C | GCC | Ala | |
| *Rv3800c* | 3041 | C | ACG | Thr | T | ATG | Met | |
| *Rv3800c* | 4937 | C | GCC | Ala | A | GAC | Asp | |
| **sz6213** | | | | | | | |  |
| *Rv0015c* | 1106 | A | CAG | Gln | G | CGG | Arg | |
| *Rv0015c* | 1109 | A | CAG | Gln | C | CCG | Pro | |
| *Rv0018c* | 1387 | C | CCG | Pro | T | TCG | Ser | |
| *Rv0039c* | 71 | G | TGC | Cys | T | TTC | Phe | |
| *Rv0078A* | 334 | G | GAG | Glu | A | AAG | Lys | |
| *Rv0201c* | 8 | T | TTG | Leu | C | TCG | Ser | |
| *Rv0201c* | 10 | G | GCA | Ala | A | ACC | Thr | |
| *Rv0201c* | 12 | A | GCA | Ala | C | ACC | Thr | |
| *Rv0201c* | 17 | A | GAG | Glu | G | GGG | Gly | |
| *Rv0201c* | 23 | A | CAC | His | C | CCC | Pro | |
| *Rv0368c* | 746 | G | CGT | Arg | A | CAT | His | |
| *Rv0444c* | 243 | G | GAG | Glu | C | GAC | Asp | |
| *Rv0452* | 373 | C | CAT | His | G | GAT | Asp | |
| *Rv0537c* | 868 | A | ACC | Thr | G | GCC | Ala | |
| *Rv0545c* | 145 | C | CCT | Pro | T | TCT | Ser | |
| *Rv0556* | 44 | T | CTC | Leu | G | CGC | Arg | |
| *Rv0565c* | 329 | G | CGC | Arg | A | CAC | His | |
| *Rv0629c* | 360 | A | GAA | Glu | C | GAC | Asp | |
| *Rv0679c* | 426 | C | AAC | Asn | G | AAG | Lys | |
| *Rv0728c* | 725 | G | CGC | Arg | A | CAC | His | |
| *Rv0747* | 2344 | A | AGC | Ser | G | GCC | Ala | |
| *Rv0747* | 2345 | G | AGC | Ser | C | GCC | Ala | |
| *Rv0758* | 515 | C | CCC | Pro | T | CTC | Leu | |
| *Rv0859* | 448 | A | AGC | Ser | G | GGC | Gly | |
| *Rv0922* | 533 | G | GGC | Gly | A | GAC | Asp | |
| *Rv0982* | 1016 | T | CTT | Leu | A | CAT | His | |
| *Rv0988* | 571 | C | CTG | Leu | G | GCG | Ala | |
| *Rv0988* | 572 | T | CTG | Leu | C | GCG | Ala | |
| *Rv0989c* | 961 | A | ATA | Ile | G | GTA | Val | |
| *Rv0995* | 67 | G | GGC | Gly | A | AGC | Ser | |
| *Rv1102c* | 194 | C | ACC | Thr | T | ATC | Ile | |
| *Rv1163* | 536 | C | CCG | Pro | G | CGG | Arg | |
| *Rv1239c* | 415 | A | AAA | Lys | G | GAA | Glu | |
| *Rv1248c* | 2290 | G | GTG | Val | A | ATG | Met | |
| *Rv1266c* | 1820 | G | CGG | Arg | A | CAG | Gln | |
| *Rv1446c* | 475 | A | AAG | Lys | C | CAG | Gln | |
| *Rv1446c* | 575 | G | CGA | Arg | C | CCA | Pro | |
| *Rv1449c* | 52 | T | TAC | Tyr | G | GAC | Asp | |
| *Rv1538c* | 841 | G | GGC | Gly | A | AGC | Ser | |
| *Rv1597* | 62 | G | GGT | Gly | A | GAT | Asp | |
| *Rv1716* | 532 | A | AGT | Ser | G | GGT | Gly | |
| *Rv1716* | 827 | T | GTC | Val | C | GCC | Ala | |
| *Rv1733c* | 204 | G | CAG | Gln | T | CAT | His | |
| *Rv1826* | 362 | C | ACA | Thr | A | AAA | Lys | |
| *Rv1837c* | 310 | G | GGT | Gly | A | AGT | Ser | |
| *Rv1894c* | 1120 | G | GCG | Ala | T | TCG | Ser | |
| *Rv2109c* | 404 | G | CGC | Arg | C | CCC | Pro | |
| *Rv2109c* | 544 | C | CGT | Arg | G | GGT | Gly | |
| *Rv2127* | 26 | G | GGC | Gly | A | GAC | Asp | |
| *Rv2187* | 299 | C | ACC | Thr | T | ATC | Ile | |
| *Rv2198c* | 132 | T | AGT | Ser | G | AGG | Arg | |
| *Rv2383c* | 2020 | G | GTG | Val | C | CTG | Leu | |
| *Rv2450c* | 377 | G | CGG | Arg | A | CAG | Gln | |
| *Rv2494* | 143 | T | GTG | Val | C | GCG | Ala | |
| *Rv2495c* | 307 | T | TAC | Tyr | G | GAC | Asp | |
| *Rv2495c* | 319 | A | ACC | Thr | G | GCC | Ala | |
| *Rv2524c* | 8311 | T | TGC | Cys | C | CGC | Arg | |
| *Rv2542* | 631 | A | ACC | Thr | G | GCC | Ala | |
| *Rv2685* | 1133 | C | GCA | Ala | G | GGA | Gly | |
| *Rv2724c* | 466 | G | GTG | Val | A | ATG | Met | |
| *Rv2861c* | 563 | A | GAG | Glu | G | GGG | Gly | |
| *Rv2931* | 3581 | T | CTA | Leu | G | CGA | Arg | |
| *Rv3015c* | 500 | T | TTC | Phe | G | TGC | Cys | |
| *Rv3051c* | 1556 | A | CAG | Gln | G | CGG | Arg | |
| *Rv3113* | 401 | G | GGA | Gly | A | GAA | Glu | |
| *Rv3236c* | 304 | A | ACG | Thr | G | GCG | Ala | |
| *Rv3296* | 1475 | A | AAG | Lys | C | ACG | Thr | |
| *Rv3317* | 160 | G | GTG | Val | C | CTG | Leu | |
| *Rv3317* | 334 | A | ACC | Thr | G | GCC | Ala | |
| *Rv3411c* | 1171 | G | GCG | Ala | A | ACG | Thr | |
| *Rv3519* | 40 | A | ACC | Thr | C | CCC | Pro | |
| *Rv3586* | 862 | A | ACG | Thr | G | GCG | Ala | |
| *Rv3715c* | 130 | G | GGT | Gly | T | TGT | Cys | |
| *Rv3729* | 805 | C | CCG | Pro | T | TCG | Ser | |
| *Rv3729* | 1548 | G | CAG | Gln | C | CAC | His | |
| *Rv3777* | 479 | T | GTC | Val | C | GCC | Ala | |
| *Rv3787c* | 754 | C | CTC | Leu | G | GTC | Val | |
| *Rv3800c* | 3041 | C | ACG | Thr | T | ATG | Met | |
| *Rv3800c* | 4937 | C | GCC | Ala | A | GAC | Asp | |
| **wx6** | | | | | | | |  |
| *Rv0018c* | 1387 | C | CCG | Pro | T | TCG | Ser | |
| *Rv0069c* | 1342 | G | GGC | Gly | A | AGC | Ser | |
| *Rv0201c* | 8 | T | TTG | Leu | C | TCG | Ser | |
| *Rv0201c* | 10 | G | GCA | Ala | A | ACC | Thr | |
| *Rv0201c* | 12 | A | GCA | Ala | C | ACC | Thr | |
| *Rv0201c* | 17 | A | GAG | Glu | G | GGG | Gly | |
| *Rv0201c* | 23 | A | CAC | His | C | CCC | Pro | |
| *Rv0511* | 1014 | G | ATG | Met | C | ATC | Ile | |
| *Rv0545c* | 145 | C | CCT | Pro | T | TCT | Ser | |
| *Rv0556* | 44 | T | CTC | Leu | G | CGC | Arg | |
| *Rv0702* | 236 | G | CGT | Arg | A | CAT | His | |
| *Rv0758* | 515 | C | CCC | Pro | T | CTC | Leu | |
| *Rv0794c* | 1000 | G | GCG | Ala | A | ACG | Thr | |
| *Rv0846c* | 1367 | G | GGC | Gly | A | GAC | Asp | |
| *Rv0859* | 448 | A | AGC | Ser | G | GGC | Gly | |
| *Rv0973c* | 235 | G | GGC | Gly | A | AGC | Ser | |
| *Rv0989c* | 961 | A | ATA | Ile | G | GTA | Val | |
| *Rv1082* | 332 | T | GTG | Val | C | GCG | Ala | |
| *Rv1239c* | 415 | A | AAA | Lys | G | GAA | Glu | |
| *Rv1245c* | 383 | A | CAT | His | G | CGT | Arg | |
| *Rv1266c* | 1820 | G | CGG | Arg | A | CAG | Gln | |
| *Rv1360* | 485 | T | GTT | Val | G | GGT | Gly | |
| *Rv1446c* | 575 | G | CGA | Arg | C | CCA | Pro | |
| *Rv1447c* | 307 | G | GAG | Glu | A | AAG | Lys | |
| *Rv1449c* | 52 | T | TAC | Tyr | G | GAC | Asp | |
| *Rv1597* | 62 | G | GGT | Gly | A | GAT | Asp | |
| *Rv1678* | 499 | A | ACG | Thr | G | GCG | Ala | |
| *Rv1716* | 827 | T | GTC | Val | C | GCC | Ala | |
| *Rv1733c* | 204 | G | CAG | Gln | T | CAT | His | |
| *Rv2033c* | 415 | C | CAT | His | T | TAT | Tyr | |
| *Rv2164c* | 1151 | G | AGG | Arg | A | AAG | Lys | |
| *Rv2450c* | 377 | G | CGG | Arg | A | CAG | Gln | |
| *Rv2483c* | 1682 | T | ATC | Ile | G | AGC | Ser | |
| *Rv2495c* | 319 | A | ACC | Thr | G | GCC | Ala | |
| *Rv2542* | 631 | A | ACC | Thr | G | GCC | Ala | |
| *Rv3317* | 160 | G | GTG | Val | C | CTG | Leu | |
| *Rv3350c* | 8542 | G | GCA | Ala | A | ACA | Thr | |
| *Rv3411c* | 178 | G | GTG | Val | A | ATG | Met | |
| *Rv3596c* | 2387 | C | CCG | Pro | T | CTG | Leu | |
| *Rv3677c* | 361 | G | GTG | Val | C | CTG | Leu | |
| *Rv3723* | 109 | G | GGA | Gly | A | AGA | Arg | |
| *Rv3800c* | 4481 | A | GAC | Asp | G | GGC | Gly | |
| *Rv3864* | 179 | C | GCG | Ala | G | GGG | Gly | |
